# An Ancient Duplication of Exon 5 in the *Snap*25 Gene Is Required for Complex Neuronal Development/Function

**DOI:** 10.1371/journal.pgen.1000278

**Published:** 2008-11-28

**Authors:** Jenny U. Johansson, Jesper Ericsson, Juliette Janson, Simret Beraki, Davor Stanić, Slavena A. Mandic, Martin A. Wikström, Tomas Hökfelt, Sven Ove Ögren, Björn Rozell, Per-Olof Berggren, Christina Bark

**Affiliations:** 1 The Rolf Luft Research Center for Diabetes and Endocrinology, Karolinska Institutet, Stockholm, Sweden; 2 Department of Neuroscience, Karolinska Institutet, Stockholm, Sweden; 3 Department of Laboratory Medicine, Karolinska Institutet, Stockholm, Sweden; The Jackson Laboratory, United States of America

## Abstract

Alternative splicing is an evolutionary innovation to create functionally diverse proteins from a limited number of genes. SNAP-25 plays a central role in neuroexocytosis by bridging synaptic vesicles to the plasma membrane during regulated exocytosis. The SNAP-25 polypeptide is encoded by a single copy gene, but in higher vertebrates a duplication of exon 5 has resulted in two mutually exclusive splice variants, SNAP-25a and SNAP-25b. To address a potential physiological difference between the two SNAP-25 proteins, we generated gene targeted SNAP-25b deficient mouse mutants by replacing the SNAP-25b specific exon with a second SNAP-25a equivalent. Elimination of SNAP-25b expression resulted in developmental defects, spontaneous seizures, and impaired short-term synaptic plasticity. In adult mutants, morphological changes in hippocampus and drastically altered neuropeptide expression were accompanied by severe impairment of spatial learning. We conclude that the ancient exon duplication in the *Snap*25 gene provides additional SNAP-25-function required for complex neuronal processes in higher eukaryotes.

## Author Summary

In evolution, duplication of genes or gene segments appears to be an efficient way to add diverse functions in more complex organisms. The SNAP-25 protein plays an important role in mediating the release of neurotransmitters and hormones. SNAP-25 exists as two variants: SNAP-25a, which is present in early development, and SNAP-25b, which is most abundant from early adulthood and onwards. We have developed mouse mutants that only express SNAP-25a, but retain normal SNAP-25 levels by replacing the SNAP-25b segment in the *Snap*25 gene with an additional SNAP-25a copy. We show that SNAP-25b is required for early postnatal development and that a balanced expression of the two proteins is a prerequisite for maintaining an operational neuronal network during adulthood. Mice that only have SNAP-25a develop seizures, and show learning deficits and anxiety. Synaptic plasticity is impaired, and structural changes are observed in areas that are connected to such behavioral functions. In man, SNAP-25 function has been linked to behavioral and neuropsychiatric disorders, including attention deficit hyperactivity disorder, ADHD. Our present findings using genetic elimination of SNAP-25b suggest that even small alterations in the regulation of the *Snap*25 gene, resulting in a disturbed balance between SNAP-25a and SNAP-25b, lead to nervous system dysfunction.

## Introduction

The evolution of more advanced organisms has required adaptation of genomes to be able to generate new gene functions. The original genome sequence analyses of different organisms surprisingly revealed that the number of genes in the human genome is only around 30,000, versus 20,000 in much simpler organisms such as the nematode *Caenorhabditis elegans* [1],[2]. However, a closer examination of these 30,000 identified human genes suggested that as many as one third of them might be false and the number of protein-coding genes in humans are close to the 19,000 found in the domestic dog [3],[4]. Instead the increased protein complexity of higher eukaryotes appears to be the consequence of the same gene encoding several functional proteins. This has been accomplished by duplication of genes or gene segments and transcriptional and post-transcriptional regulation. The major contribution to protein diversity is alternative splicing and some 40–60% of all mammalian genes generate more than one protein [5],[6]. Interestingly, as many as 10% of all genes in mammals contain tandemly duplicated exons, suggesting that exon duplication followed by functionally diverging mutations have been a fast and successful evolutionary mechanism to increase protein variety [7],^8^. Duplicated exons are often subjected to mutually exclusive alternative splicing, incorporating only one of the two exons in the resulting polypeptide [8].

Regulated membrane fusion forms the basis for synaptic transmission but is also fundamental for appropriate release of hormones and modulatory neuropeptides [9]–[13]. One of the final steps prior to vesicle fusion with the plasma membrane is the formation of a trans-membrane *s*oluble *N*-ethylmaleimide-sensitive factor (NSF) *a*ttachment protein *re*ceptor (SNARE) complex [14],[15]. This specialized SNARE complex is bridging vesicles to the plasma membrane during regulated exocytosis and consists of three compartmentally defined proteins: The vesicle-associated VAMP2/synaptobrevin, a protein with a plasma membrane anchor syntaxin 1a, and the cytoplasmic *s*y*n*aptosomal-*a*ssociated *p*rotein of 25 kD, SNAP-25, that associates with membranes through palmitoylation. The three SNARE proteins are held together by strong protein-protein interactions, whereby the cytoplasmic domains form a four α-helix coiled-coiled bundle [16]. The detailed mechanisms mediating regulated exocytosis are still not fully elucidated but the current hypothesis is that SNARE proteins operate at the actual fusion event and have intrinsic capabilities to perform membrane fusion [17],[18]. A possibility is that the SNARE proteins are candidates for adjusting thresholds for synaptic plasticity in more advanced neuronal systems. They form the central fusogenic core at the plasma membrane. It is notable that the number of SNARE proteins, and alternative isoform variants, have increased through evolution and their expression is strictly regulated both anatomically and temporally [19]–[23].

In higher vertebrates SNAP-25 is expressed as two developmentally regulated and complementary distributed splice variants termed SNAP-25a and SNAP-25b [23]. The alternative splicing is an obligate choice between two closely spaced tandemly arranged exon 5 sequences, and nine of 206 amino acids in the two polypeptides differ. The alternative splicing modifies a domain of the SNAP-25 protein spanning a quartet of cysteine residues that are substrates for post-translational palmitoylation and required for membrane targeting [24],[25]. In mouse brain, SNAP-25a precedes SNAP-25b expression during development, but by the second postnatal (PN) week SNAP-25b becomes the major splice variant, concomitantly with a dramatic increase in SNAP-25 expression [26]. In fact, in adult mouse brain the SNAP-25b transcript represents more than 90% of total SNAP-25 mRNA [26]. Targeted disruption of the mouse *Snap25* gene has demonstrated that complete removal of SNAP-25 results in total absence of evoked neuroexocytosis and embryonic lethality [27]. Separate overexpression of the two SNAP-25 isoforms in embryonic adrenal chromaffin cells from these SNAP-25 knock-out (KO) mice showed that the SNAP-25b isoform had a higher capability to stabilize the pool of primed vesicles than SNAP-25a, since the burst of Ca^2+^-evoked catecholamine release differed [28]. Recently, genome-wide scans and linkage analysis have indicated an association between polymorphisms in the human SNAP-25 gene and vulnerability to develop attention deficit hyperactivity disorder, ADHD [29],[30]. In humans, different SNAP-25 alleles also demonstrate inheritance correlated to intelligence [31],[32].

To specifically explore the physiological importance of the exon 5 duplication in the *Snap25* gene we used a novel approach by developing SNAP-25b KO/SNAP-25a knock-in mouse mutants. The exon 5b was genetically eliminated and replaced with a second supplementary exon 5a, to maintain alternative splicing and normal expression levels, but allowing only SNAP-25a to be expressed. The SNAP-25b deficient mouse mutants exist in two versions, *neo*-containing with a *Tkneo* marker retained and *neo*-excised with the selection gene removed. Based on results from electrophysiological, behavioral and immunohistochemical experiments we conclude, that under physiological conditions, mice deficient in SNAP-25b have developmental defects, impaired short-term synaptic plasticity, a seizure-prone phenotype and malfunctioning cognitive performance. We suggest that the ancient duplication in the *Snap25* gene followed by regulated alternative splicing between two similar but distinct exon 5 sequences is required for accurate synaptic function during development. Furthermore, a balanced expression of the two isoforms is a prerequisite for maintaining an operational neuronal network also during adulthood in advanced organisms.

## Results

### Mouse Mutants Not Expressing SNAP-25b

**Figure 1:**
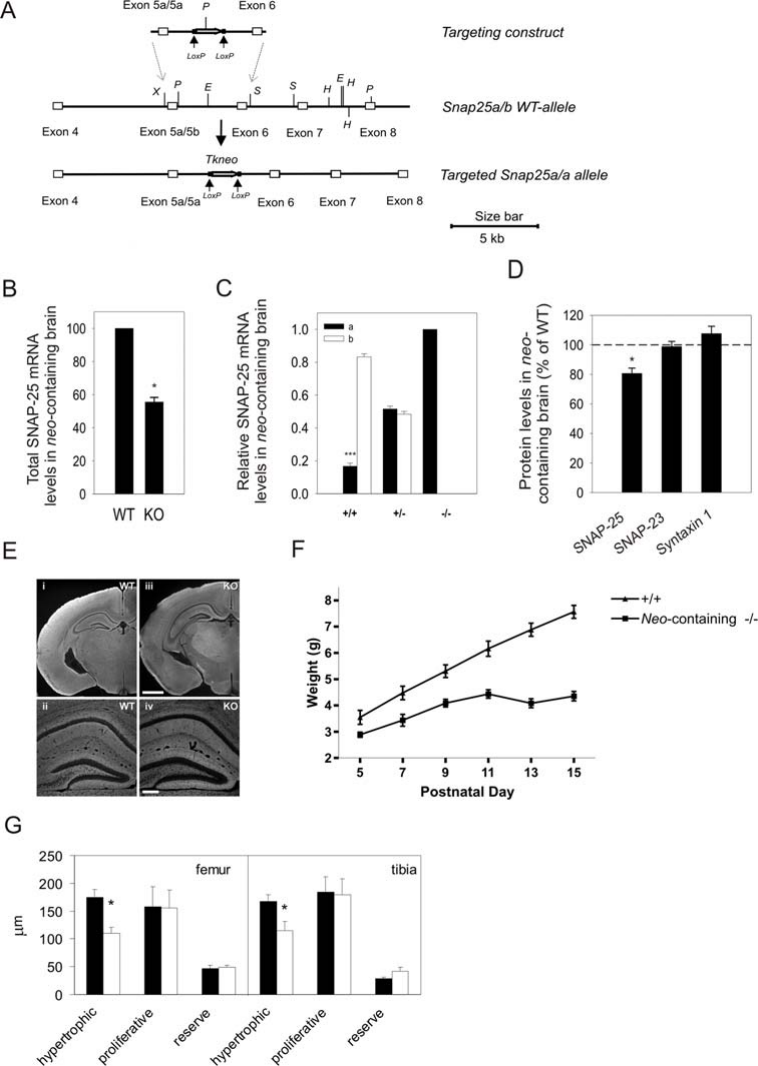
Generation of SNAP-25b Deficient Mouse Mutants. (A) Schematic diagram demonstrating the targeting construct and the development of a modified *Snap25* allele with eliminated SNAP-25b expression. The mouse genomic sequence was derived from the WT *Snap25* gene and addition of an additional exon 5a was performed using PCR. A *Tkneo* selection gene, surrounded by loxP repeats, was inserted at the *EcoRI* (E) site located downstream of the two tandemly arranged exons 5a and upstream of exon 6. Letters denote restriction sites: E, *EcoRI*; H, *HindIII*; P, *PstI*; S, *SacI*; X, *XbaI*. (B) Total SNAP-25 mRNA level in mouse brain at PN14 was investigated by semi-quantitative RT-PCR. Levels of SNAP-25 mRNA, quantified relative to GAPDH mRNA levels, were determined in *neo-*containing SNAP-25b deficient mutants (KO) and compared to WT littermates. SNAP-25 mRNA in *neo-*containing SNAP-25b deficient mutant brain was significantly reduced to 55.5±2.7% compared to WT (mean±S.E.M., n = 6 mice for each genotype, each sample repeated three times, *p = 0.0312, Wilcoxon's signed-rank test). (C) A semi-quantitative RT-PCR restriction enzyme assay was used to determine relative levels of SNAP-25a and SNAP-25b isoform mRNAs in WT (+/+), *neo-*containing heterozygous (+/−) and *neo-*containing homozygous (−/−) mutant brains at PN14. A *PvuI* restriction site exclusive for exon 5a and a *StyI* site only present in exon 5b was used to determine relative levels of SNAP-25a and SNAP-25b. WT mice had 16.7±1.9% SNAP-25a mRNA, *neo-*containing heterozygous mutants 51.6±1.7% SNAP-25a mRNA, and homozygous SNAP-25b deficient mutants only, the SNAP-25a mRNA isoform (n = 5 mice of each genotype, run in two replicates, mean±S.E.M., ***p<0.0001, Student's t-test). (D) Western blotting was used to analyze synaptic protein levels in brain at PN14-15, standardized against α-tubulin. The level of SNAP-25 protein in *neo*-containing SNAP-25b deficient mutants was 80.7±3.6% compared to WT (mean±S.E.M., *p = 0.0312, Wilcoxon's signed-rank test), whereas expression of SNAP-23 protein was 98.9±3.5% (*p = *1, n.s.) and that of syntaxin 1 protein 107.6±4.9% (*p = *0.4375, n.s.), compared to WT animals. The levels of SNAP-25 protein differed significantly in *neo*-containing SNAP-25b deficient mutants compared to WT littermates, but not the levels of SNAP-23 and syntaxin 1 proteins (n = 6 mice of each genotype, run in three replicates). (E) Representative images of SNAP-25 immunoreactivity in coronal sections at low magnification (i, iii) and in the hippocampus at higher magnification (ii, iv). No obvious differences in immunoreactivity pattern was observed between *neo*-containing SNAP-25b deficient (iii, iv) and WT (i, ii) mice at PN14 (n = 2 mice of each genotype, and three levels were analyzed from each animal). Identical microscope settings were used for WT and KO (*neo*-containing SNAP-25b deficient mutants) images. Scale bar = 1 mm for the low magnification and 200 µm for the high magnification figures. (F) Weight curves of homozygous *neo-*containing SNAP-25b deficient mutants compared to WT littermates between PN5 to PN15. Body weight gain was significantly reduced in young *neo-*containing SNAP-25b deficient mutants when compared to WT (***p<0.0001, two-way repeated measures ANOVA, n = 7 mice of each genotype). (G) Bone growth is affected in *neo-*containing SNAP-25b deficient mice. Mean±S.E.M. thickness of the hypertrophic, proliferative, and reserve zones in femur and tibia in WT (black bars) and *neo-*containing SNAP-25b deficient (white bars) PN14 mice. *p<0.05 (n = 4). Data was analyzed with unpaired Student's t-test.

In the *Snap25* gene, exon 5a and 5b are closely spaced and differ in only nine of 39 amino acids [23],[26]. To develop mouse mutants that only express SNAP-25a, a gene targeting vector with an additional exon 5a sequence, replacing the mouse exon 5b sequence, was generated (Figure 1A). The vector construct spanning two exon 5a sequences arranged in tandem and exon 6 from the mouse *Snap25* gene also contained a *Tkneo* selection cassette flanked by loxP recombination sites (Figure 1A). Only the exon 5b sequence was changed to encode the exon 5a amino acids, and the original splicing signals for expression of the downstream exon 5 were kept intact. Three independent mouse lines were backcrossed on C57BL/6NCrl (B6) mice for at least ten generations, thus establishing fully congenic strains. After intercrossing of heterozygous animals we found a mendelian distribution of genotypes (24% homozygous mutants of 125 mice), strongly indicating that our introduced genetic changes did not give rise to a prenatal lethal phenotype. However, after the second PN week all homozygous SNAP-25b deficient mice exhibited neurological defects and were sacrificed.

Thus, we have developed a SNAP-25b deficient mouse mutant by replacing exon 5b with an additional exon 5a equivalent, thereby preventing expression of SNAP-25b but not the alternative splicing.

### SNAP-25 in *Neo*-Containing SNAP-25b Deficient Mice

We previously demonstrated that targeted insertion of a *Tkneo* selection cassette in the *Snap25* gene impaired alternative splicing and repressed total gene expression [33]. Initially, we therefore investigated the level of SNAP-25 mRNA and protein expression in brain of *neo*-containing SNAP-25b deficient mutants and wild-type (WT) littermates at PN14. Semi-quantitative RT-PCR analysis demonstrated that *neo*-containing SNAP-25b deficient mice expressed approximately 50% of the SNAP-25 mRNA levels present in WT littermates (Figure 1B). In order to determine the relative ratio of SNAP-25a and SNAP-25b mRNA expression in brain at PN14, isolated RNA from homozygous and heterozygous SNAP-25b deficient mutants and WT littermates was subjected to an RT-PCR assay based on the presence of exclusive restriction enzyme sites in exons 5a and 5b. At PN14, SNAP-25b levels were five times higher than SNAP-25a in WT mice, heterozygous *neo*-containing mutants had equal amounts of both SNAP-25 mRNA isoforms, while homozygous *neo*-containing SNAP-25b deficient mutants only expressed SNAP-25a (Figure 1C).

Western blotting of protein homogenates from PN14-15 homozygous *neo*-containing SNAP-25b deficient mouse brains revealed that SNAP-25 protein levels were also lower in mutants when compared to WT littermates (Figure 1D). SNAP-25b deficient mice expressed approximately 80% of normal SNAP-25 protein levels. Thus, there was no direct correlation between SNAP-25 mRNA and protein levels (compare Figures 1B and 1D). The protein levels of the cellular SNAP-25 homolog, SNAP-23, and the binding partner to SNAP-25, syntaxin 1, were not significantly different in mutants compared to control animals (Figure 1D). Immunohistochemical analysis of SNAP-25 showed a similar appearance in homozygous *neo*-containing SNAP-25b deficient mutant and WT mouse brain at PN14, and no obvious pathological changes were observed (Figure 1E).

Together, our results demonstrate that homozygous *neo*-containing SNAP-25b deficient mouse mutants only express SNAP-25a and that total SNAP-25 mRNA and protein levels are reduced. Unexpectedly, despite that *neo*-containing SNAP-25b deficient mutants express only 50% of normal SNAP-25 mRNA levels, protein levels of SNAP-25 are only reduced to 80% of WT littermates.

### *Neo*-Containing SNAP-25b Deficient Mutants Die Young

*Neo*-containing SNAP-25b deficient mutants exhibited a severe phenotype that included a reduced gain in body weight after the first PN week (Figure 1F). The growth-deficiency was not due to an inability of mutants to feed properly, as dissection revealed stomachs filled with milk (data not shown). To further investigate developmental defects, bone growth was analyzed in PN14 mutants and WT littermates. Bone development was determined by measuring the hypertrophic, proliferative and reserve zones in hind limb sections (Figure 1G). In both the femur and tibia, the hypertrophic zones were significantly reduced in homozygous *neo*-containing SNAP-25b deficient mutants when compared to WT littermates. The proliferative and reserve zones were not significantly different (Figure 1G). Around PN10 homozygous SNAP-25b deficient mice were easily identified by their smaller size and extreme activity with hyperactive episodes. From PN11, *neo*-containing SNAP-25b deficient mutants exhibited frequent episodes with tremors and seizure activity and were therefore sacrificed at PN14-17. The early PN development also appeared postponed, indicated by that eye opening occurred approximately one day later compared to WT littermates and that the SNAP-25b deficient mutants also demonstrated inability to respond to sound concurrently in time when WT littermates acquired that ability. Heterozygous *neo*-containing SNAP-25b mutants were indistinguishable from WT littermates during early PN development.

Our results show, that homozygous *neo*-containing SNAP-25b deficient mice, lacking SNAP-25b expression and having a moderate reduction in total levels of SNAP-25 protein, exhibit severe developmental and behavioral defects.

### Removal of the Selection Gene, *Tkneo,* Reduces the Severity of the Phenotype

**Figure 2:**
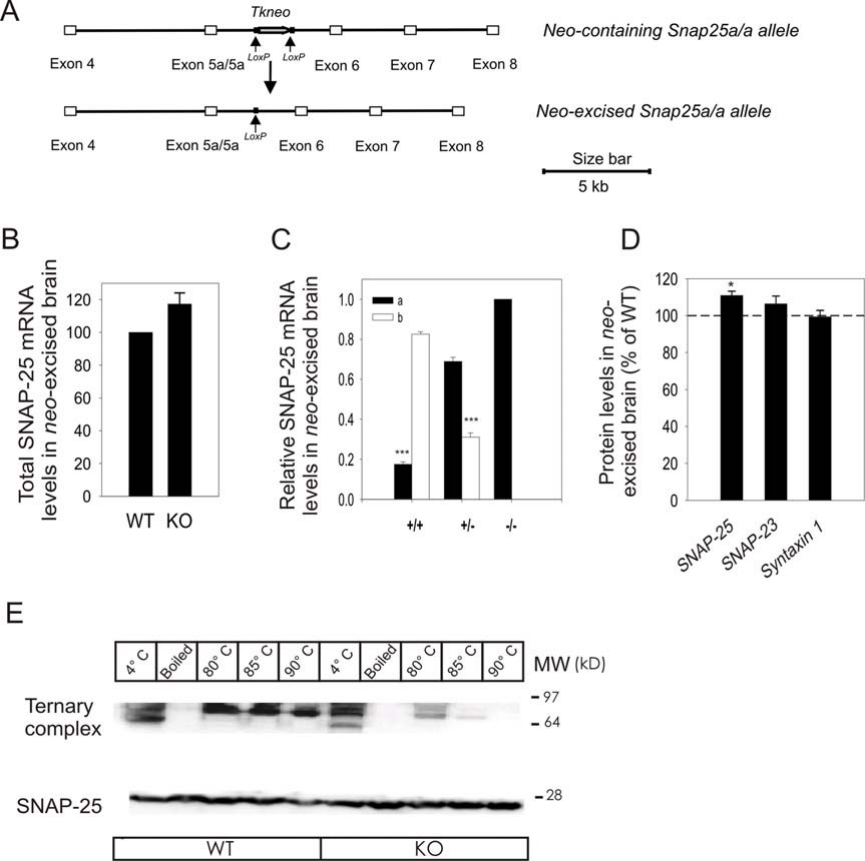
*In vivo* Excision of the *Tkneo* Gene Results in Altered Gene Expression. (A) A schematic diagram demonstrating the *in vivo* excision of the *Tkneo* gene. Heterozygous *neo-*containing SNAP-25b deficient females were mated with heterozygous Prm1Cre males. Male offspring carrying both the mutation in the *Snap25* gene and the Cre transgene were mated with B6 females. *In vivo* excision was demonstrated by PCR, and the Cre transgene was crossed out from the confirmed *neo-*excised SNAP-25b deficient mice. (B) To investigate total SNAP-25 mRNA levels in mouse brain of *neo-*excised SNAP-25b deficient mutants at PN14-15, the same radioactive semi-quantitative RT-PCR assay as for *neo-*containing mouse mutants (see Figure 1B) was used. Total SNAP-25 mRNA levels (SNAP-25a + SNAP-25b) in *neo-*excised SNAP-25b deficient mice brain at PN14-15 were 117.3±6.8% of WT (n = 6 mice for each genotype, repeated three times each, mean±S.E.M., *p = 0.0625, Wilcoxon's signed-rank test). (C) SNAP-25a/SNAP-25b mRNA ratio at PN14 were for WT littermates 17.5±1.2% and 82.5±1.2%, respectively (mean±S.E.M., ***p<0.0001 Student's t-test), in heterozygous *neo-*excised SNAP-25b mutants 68.9±2.1% SNAP-25a and 31.1±2.1% SNAP-25b (***p<0.0001), and in homozygous *neo-*excised SNAP-25b deficient mice 100% SNAP-25a (n = 5 mice for each genotype, repeated twice). (D) Protein levels in brain at PN14-15. In *neo-*excised SNAP-25b deficient mice, there was 110.9±2.3% (*p = 0.0312) SNAP-25 protein, 106.4±4.2% (*p = *0.3125, n.s) SNAP-23, and 99.4±3.5% (*p = *0.8438, n.s.) syntaxin 1 relative to WT expression levels (n = 6 mice for each genotype, run in three replicates, mean±S.E.M., Wilcoxon's signed-rank test). (E) Immunoblot demonstrating temperature dependence and stability of the ternary SDS resistant SNARE complex in *neo*-excised SNAP-25b deficient (KO) and WT mouse brain cortex. The samples separated in lanes 1–5 (from left to right) are from adult WT brain cortex, and the preparations in lanes 6–10 are from *neo*-excised SNAP-25b deficient mutants (KO only expressing SNAP-25a). The high molecular weight ∼97 kD complex was identified as SNAP-25 in ternary SNARE complex, while the band migrating as ∼28 kDa is free SNAP-25 protein. KO brain homogenates heated to 80°C showed 44±8% SNAP-25 still present in ternary complex compared to 4°C samples, significantly lower than for SNAP-25 in WT brain (71.2±7.2, *p = 0.01). At 85°C and 90°C, the difference in percentage of SNAP-25 remaining in complex between WT and KO was as following: 59.8±7 and 36.9±3.4 (*p = 0.018), and 48.3±12.3 and 4.5±2.2 (**p = 0.0081), respectively. All experiments were repeated at least three times. Results were analyzed with unpaired Student's t-test and summarized as mean±S.E.M.

The reduced SNAP-25 expression in *neo*-containing SNAP-25b deficient mutants could be due to the presence of the *Tkneo* selection gene. Therefore, *neo*-containing heterozygous SNAP-25b deficient mutants were crossbred with a global Cre transgene [34]. The *Tkneo* gene was excised and the Cre transgene was thereafter crossed out from the *neo*-excised SNAP-25b deficient mutants (Figure 2A).

*Neo*-excised SNAP-25b mutants were backcrossed onto a B6 background for more than ten generations. Intercrosses using heterozygous breeding pairs indicated the expected mendelian distribution (28% homozygous mutants born out of 222 mice). Unlike *neo*-containing homozygous SNAP-25b deficient mutants, the growth of *neo*-excised homozygous mutants between PN5 and 15 was not severely affected. No difference in body weight was observed between mutants and WT littermates when genders were mixed. However, when females were analyzed separately they demonstrated a small but significant reduction in body weight (Text S1, Figure S1A and S1B). After the first PN weeks the body weights were normalized and no significant differences were observed in adult females (data not shown). *Neo*-excised SNAP-25b deficient mutants also demonstrated spontaneously occurring seizures although less frequent and not prior to young adulthood.

In conclusion, *in vivo* excising of *Tkneo* from the targeted *Snap25* gene rescues the most severe developmental defects observed in homozygous *neo*-containing SNAP-25b deficient mice.

### SNAP-25b Deficient Mutants Exhibit No Reduction in SNAP-25 Expression

Contrary to homozygous *neo*-containing animals, *neo*-excised SNAP-25b deficient mutants showed SNAP-25 mRNA levels similar to WT littermates at PN14-15 (Figure 2B). SNAP-25b mRNA in WT mice was, as expected, five times higher than SNAP-25a (Figure 2C). In heterozygous *neo*-excised mutants the relative SNAP-25a/SNAP-25b mRNA ratio was 2∶1, while SNAP-25b was absent in homozygous *neo*-excised mutants. In *neo*-excised mutants SNAP-25 protein expression was moderately but significantly increased to 111% compared to WT (Figure 2D). SNAP-23 and syntaxin 1 protein levels were not altered from levels observed in control littermates.

Our results demonstrate that removal of *Tkneo* restores SNAP-25 expression and moderately increases total SNAP-25 protein levels in brain of homozygous *neo*-excised SNAP-25b deficient mutants.

### SNARE Complexes from *Neo*-Excised SNAP-25b Deficient Mutants Are Less Stable Than Those from WT Mouse Brain

In adult brain of WT mice, both splice variants of SNAP-25 are expressed but SNAP-25b is the predominant isoform comprising 90–95% of total SNAP-25 mRNA [26]. To investigate if the stability of SNARE complexes was altered in the brain of our *neo*-excised SNAP-25b deficient mutants compared to WT mice, we analyzed SDS-resistant SNARE complexes at different temperatures (Figure 2E). Adult brain tissue homogenates only containing SNAP-25a (*neo*-excised SNAP-25b deficient mutants, KO) or predominantly SNAP-25b (WT) were incubated at different temperatures, either 4°C or heated, prior to gel electrophoresis and Western blotting. In non-heated samples, several immunoreactive bands were observed in SNAP-25b deficient and in WT brain homogenates (Figure 2E). Except for a SDS resistant complex observed at ∼97 kD, identified as the ternary SNARE complex, additional intermediate complexes were detected. At 4°C and stepwise increasing temperatures (70°C–90°C) the ratio of the quantified protein band migrating at ∼28 kDa (free SNAP-25) to higher molecular weight SNAP-25 containing complexes indicated that the stability of SNARE complexes was reduced in SNAP-25b deficient mouse brain. Quantification of immunoreactive bands from WT and *neo*-excised SNAP-25b deficient mutants mice showed that the percentage of total SNAP-25 still present in the ternary complex differed significantly at 80°C, 85°C and 90°C [at 80°C, 71.2±7.2% and 44.0±8.0% (*p<0.05); at 85°C 59.8±7 and 36.9±3.4 (*p<0.05), and at 90°C 48.3±12.3 and 4.5±2.2 (**p<0.01)]. The ternary complex almost disappeared in SNAP-25b deficient brain preparations treated at 90°C while the WT samples still showed a strong immunoreactive signal containing 48.3% of total SNAP-25 in non-disassociated complex (Figure 2E). Boiling of the samples resulted in the loss of all immunoreactive bands except the monomeric SNAP-25 in both *neo*-excised SNAP-25b deficient and WT mice.

Our results demonstrate that SNARE complexes isolated from brain of adult *neo*-excised SNAP-25b deficient mutants are less stable than SNARE complexes from adult WT mice.

### *Neo*-Excised SNAP-25b Deficient Mutants Demonstrate a Reduction of Facilitated Release

**Figure 3:**
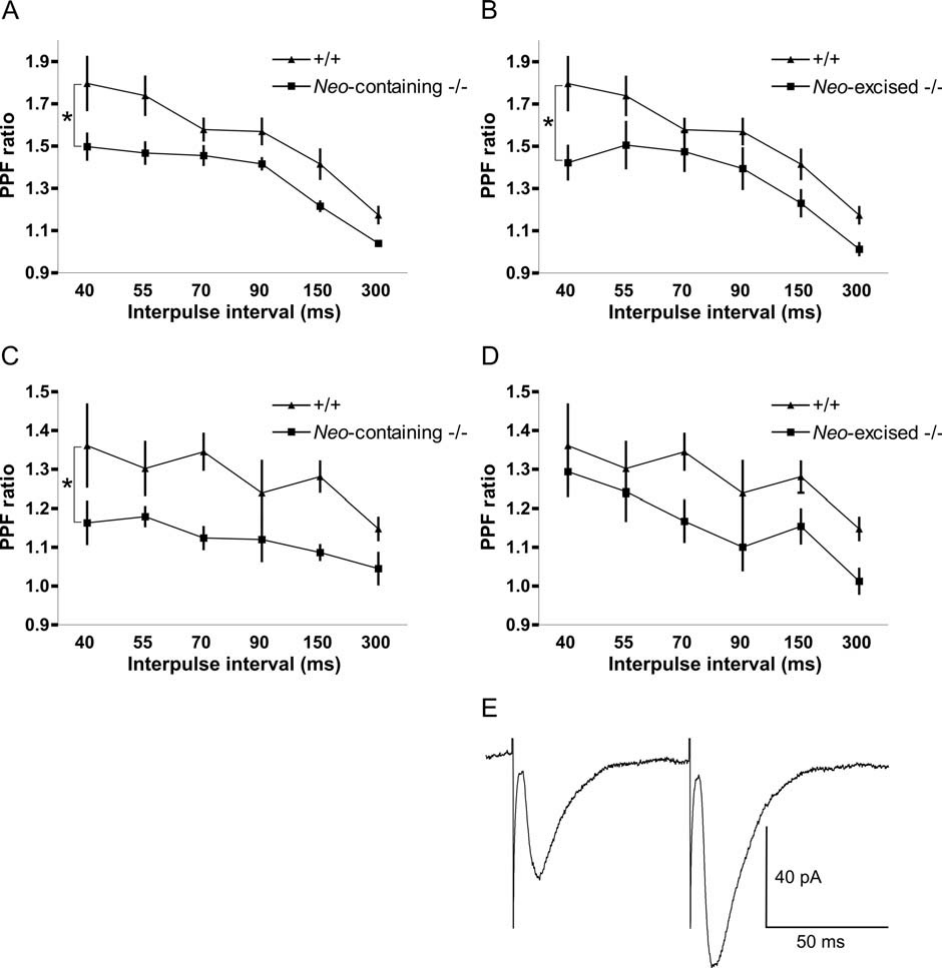
Short-term Plasticity Analyzed in SNAP-25b Deficient Mouse Mutants. Paired-pulse facilitation (PPF) is reduced at Schaffer collateral-CA1 pyramidal cell synapses in both *neo*-containing and *neo*-excised SNAP-25b deficient slices. PPF (PPF ratio = EPSC_2_/EPSC_1_) was recorded in the whole-cell voltage clamp mode as a function of the interpulse interval (IPI) at two different frequencies in slices from PN12-16 animals. Error bars indicate S.E.M. (A) Reduced PPF at 0.2 Hz in *neo*-containing SNAP-25b deficient mutants compared to WT mice (*p<0.05). (B) Reduced PPF at 0.2 Hz in *neo*-excised SNAP-25b deficient mice compared to WT (*p<0.05). (C) Reduced PPF at 0.5 Hz in *neo*-containing SNAP-25b deficient mutants compared to WT (*p<0.05). (D) PPF at 0.5 Hz in *neo*-excised SNAP-25b deficient mice compared to WT (*p = *0.085 n.s.). (E) An example of a typical PPF trace of Schaffer collateral-CA1 EPSCs from a *neo*-containing SNAP-25b deficient mutant mouse recorded at 70 ms IPI. Statistical comparisons of PPF ratios between WT and SNAP-25b deficient mice were made with two-way repeated measurements analysis of variance, ANOVA.

SNAP-25 is essential for evoked synaptic transmission [27] and its activation is dependent on cytoplasmic free Ca^2+^-concentrations [35]. The different stability of SNARE complexes found in *neo*-excised SNAP-25b deficient mutants compared to WT mice (Figure 2E) and that SNAP-25b demonstrates an increased association with plasma membrane fractions compared to SNAP-25a (see Text S1, Figure S2) suggest that SNAP-25a and SNAP-25b are differently associated with SNARE complexes close to, or immediately upstream of fusion. Therefore, we compared paired-pulse facilitation (PPF, Figure 3E) of AMPA receptor-mediated synaptic transmission in young WT, *neo*-containing and *neo*-excised SNAP-25b deficient mice (KO) at the Schaffer collateral-CA1 pyramidal neuron synapses of the hippocampus [36],[37]. Two stimulus frequencies were used, 0.2 and 0.5 Hz and the interpulse interval (IPI) varied between 40 and 300 ms. *Neo*-containing SNAP-25b deficient mouse mutants (n = 8 cells in 6 mice) showed a reduction (*p<0.05) in PPF at 0.2 Hz compared to WT mice (n = 9 cells in 5 mice, Figure 3A). A decrease was also observed at 0.5 Hz for these mice (*p <0.05, n = 7 cells in 7 mice) compared to WT littermates (n = 9 cells in 7 mice, Figure 3C). Furthermore, *neo*-excised SNAP-25b deficient mouse mutants demonstrated a clear reduction in PPF at 0.2 Hz (*p <0.05, SNAP-25b KO: n = 9 in 4 mice; WT: n = 9 cells in 5 mice, Figure 3B) but not at 0.5 Hz (*p* = 0.085; n.s., SNAP-25b KO: n = 9 cells in 6 mice; WT: n = 9 cells in 7 mice, Figure 3D) compared to WT mice.

Finally, we compared the PPF-ratios obtained from homozygous *neo*-containing and *neo*-excised SNAP-25b deficient animals. No significant differences were found at either 0.2 (*p* = 0.92, n.s.) or 0.5 Hz (*p* = 0.48, n.s.). All recorded neurons were responsive to the presynaptic stimulation and there was no obvious rundown or difference in baseline responses, in contrast to what is seen in complete SNAP-25 KO mice [38].

Our results demonstrate that absence of SNAP-25b reduces PPF at Schaffer collateral-CA1 synapses in PN12-16 mice during low-frequency stimulation.

### *Neo*-Excised SNAP-25b Deficient Mouse Mutants Demonstrate Cognitive Impairment

**Figure 4:**
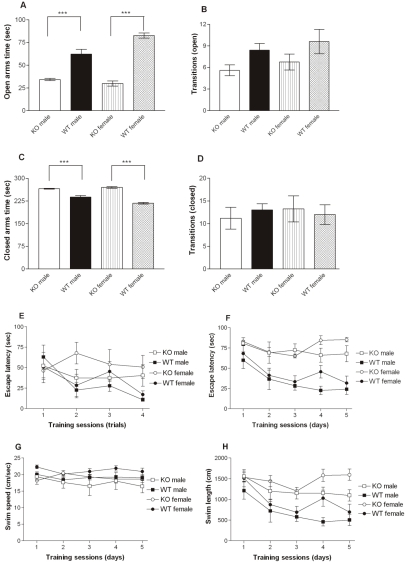
Elevated Plus Maze Test and the Morris Water Maze Task. (A-D) Anxiety-like behavior in the elevated plus maze test. Time spent on open arms (A) (***p<0.001), time spent on closed arms (C) (***p<0.001), and number of crossings to the open (B) and closed arms (D). Each value represents the mean (±S.E.M.) from the four groups of animals. The homozygous *neo*-excised SNAP-25b deficient females (n = 4), the *neo*-excised SNAP-25b deficient male mice (n = 5), WT females (n = 5), and WT males (n = 5). (E–H) Spatial learning in the Morris water maze. Latency of the four experimental groups to locate a visible platform placed randomly within the water tank (E). The latency to find the hidden platform (F) as well as swim speed (G) and swim length (H) are shown. The results are presented as mean values±S.E.M. (average over four trials per session), from *neo*-excised SNAP-25b deficient mutant females (n = 4), SNAP-25b deficient males (n = 5), WT females (n = 5), and WT males (n = 5). The mutants differed from the control groups during the five days of training, both regard to escape latency and swim distance (*p*<0.001). Data from the behavioral testing was analyzed by non-parametric statistics using Kruskal-Wallis ANOVA followed by the Mann-Whitney U test as the post-hoc test.

In view of the above findings, we investigated the potential role of SNAP-25b in behavioral functions partly related to changes in long-term plasticity in the hippocampus and amygdala. The mice were examined in the elevated plus maze, a behavioral test primarily designed for analyzing anxiety-related behavior. Homozygous n*eo*-excised SNAP-25b deficient mutants spent less time in the open arms compared to the corresponding control group (***p<0.001, Figure 4A) and more time in the closed arms during the 5 min observation period (***p<0.001, Figure 4C). The total number of arm entries did not differ between mutants and control animals, which indicates that there were no differences in overall motor activity between the two groups (Figures 4B and 4D).

Acquisition and retention of spatial learning, which involve hippocampal mechanisms, were studied in the Morris water maze task. In the pre-training phase, there was no overall significant effect of genotype (*p* = 0.09, n.s.) in the latency to navigate to the visible platform compared to the corresponding control group (Figure 4E). However, a subsequent post-hoc analysis showed that homozygous *neo*-excised SNAP-25b female mice had a significant higher latency compared to the control mice on day 2 and on day 4 (*p*<0.05). Spatial acquisition was examined during five days of training and revealed highly significant differences between the groups with regard to escape latency (*p*<0.001, Figure 4F), indicating that *neo*-excised SNAP-25b deficient mice were impaired in their ability to acquire the spatial learning task compared to WT controls. In addition, *neo*-excised SNAP-25b deficient mutants had a longer swim distance compared to controls (*p*<0.001, Figure 4H). There were no overall differences with regard to swim speed between *neo*-excised SNAP-25b deficient mutants and the control groups (Figure 4G). The analysis of percent swimming along the wall in the water tank, e.g. thigmotaxis revealed that both *neo*-excised SNAP-25b deficient males and females displayed a much higher thigmotactic behavior than the control animals (*p*<0.001).

Young *neo*-containing SNAP-25b deficient mutants demonstrated periods with profound hyperactivity in their home cages, a behavior not observed in *neo*-excised SNAP-25b deficient mutants. Locomotor activity in adult *neo*-excised SNAP-25b deficient mutants was investigated in computerized locomotor cages. Analyses of spontaneous locomotor activity during the 60 min recording revealed that for all measures, e.g. motility, locomotion and rearing, the *neo*-excised SNAP-25 deficient mutants had lower (*p*<0.01) locomotor activity than WT controls. Female mutants demonstrated a low activity level already during the first 10 min of recording, i.e. during the initial exploration of the locomotor cage (data not shown).

In summary, *neo*-excised SNAP-25b deficient mice, irrespective of gender demonstrate a higher anxiety index, a severe impairment in spatial learning with females being most severely affected, and a lower locomotor activity when compared to WT controls.

### Pathological Changes Develop in Hippocampus by Age

**Figure 5:**
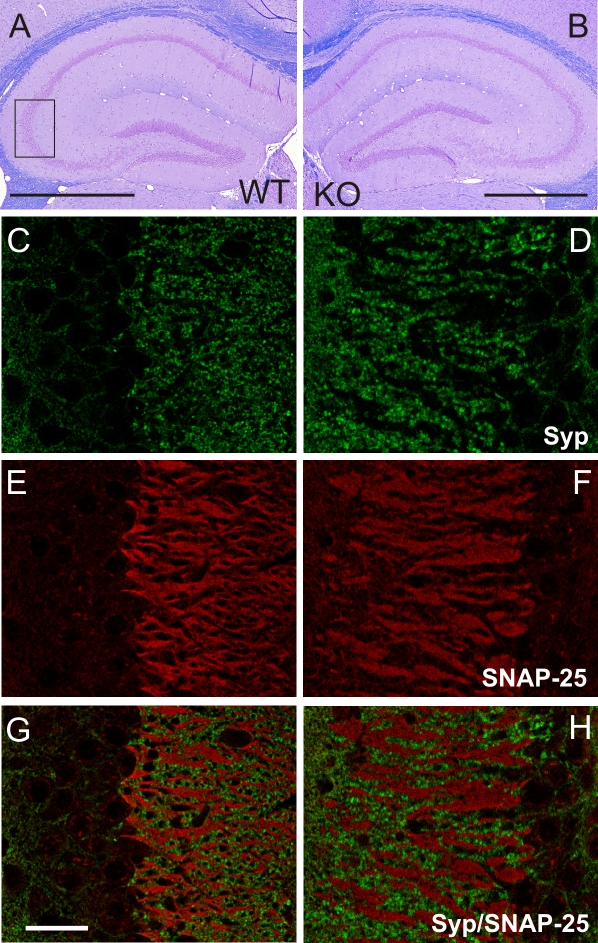
Brain Pathology Develops in Adult *Neo*-excised SNAP-25b Deficient Mice. (A–B) Cresyl violet stained coronal sections of adult WT (A) and *neo*-excised SNAP-25b deficient (B) mice at the level of the hippocampus. The box indicates the CA3 area that is shown at higher magnification in (C–H). Scale bars = 1 mm. (C–H) Representative images of synaptophysin (green) and SNAP-25 (red) (E,F) immunoreactivity in the hippocampus of 4-month-old WT (left column) and *neo*-excised SNAP-25b deficient mutants (right column), and double immunofluorescence images (G,H). Scale bar = 50 µm (n = 3 animals of each genotype, each brain analyzed at least at three different levels).

In young *neo*-containing and *neo*-excised SNAP-25b deficient mouse mutants no obvious difference in structural and morphological appearance of the brain was observed by histological and immunohistochemical analysis (Figure 1E, and data not shown). However, in the stratum lucidum (SLu) of the CA3 region of 4-month-old *neo*-excised SNAP-25b deficient mutants, SNAP-25-positive (+) mossy fibers had expanded and formed large bundles (compare Figure 5E and 5F), separating the synaptophysin^+^ nerve endings into island-like structures (Figure 5G and 5H). These morphological differences observed with immunohistochemistry were also evident in *neo*-excised SNAP-25b deficient mouse mutants at 2 months of age and appeared to progress with age. The CA1 and cerebellum were unaffected at all ages analyzed (data not shown). Thus, the absence of SNAP-25b results in progressing pathological changes in brain areas important for cognitive functions.

### Change in Expression of Neuropeptides, BDNF, and Doublecortin

Epileptic activity causes dramatic changes in peptide expression in the hippocampal formation (HF), in particular affecting the granule cell-mossy fiber system, termed ‘epilepsia peptidergic profile’ [39]–[42]. Since we observed seizure activity in our mouse mutants, we examined with immunohistochemistry the expression of cholecystokinin (CCK), neuropeptide Y (NPY) and brain-derived neurotrophic factor (BDNF) in the HF of 8-week-old *neo*-excised SNAP-25b deficient mutants and WT littermates. Numbers of doublecortin (DCx)^+^ migrating neuronal precursor cells were also inspected, as seizures may increase neurogenesis.

**Figure 6:**
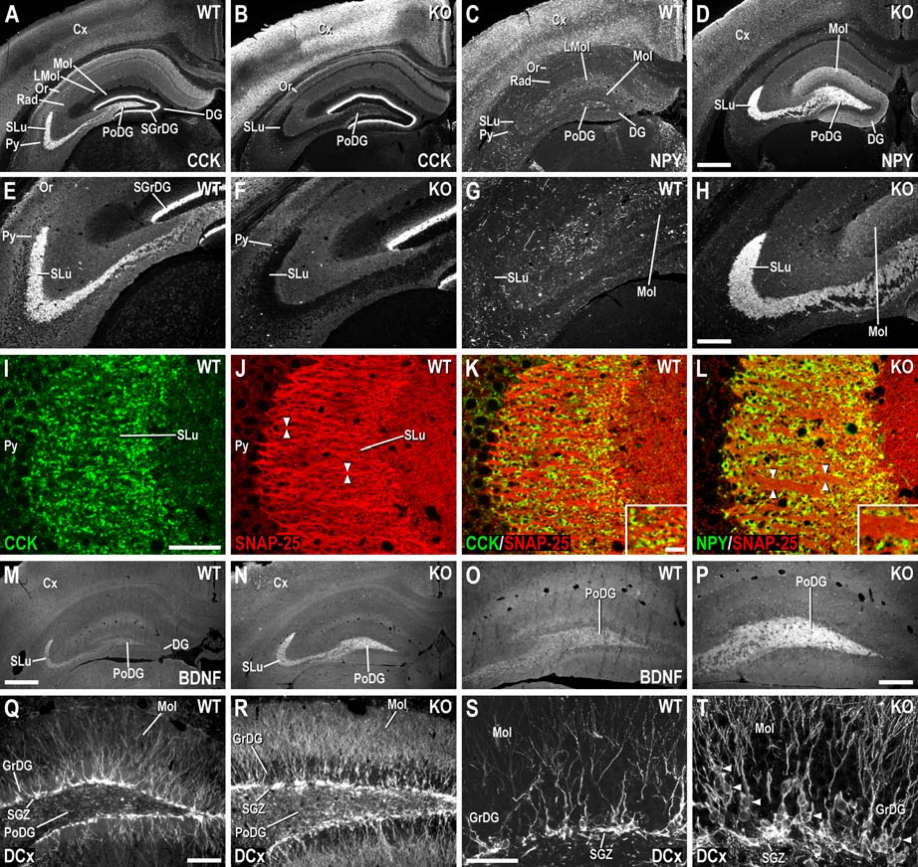
SNAP-25, CCK, NPY, BDNF, and DCx Immunoreactivity in the Hippocampal Formation of Adult *Neo*-excised SNAP-25b Deficient and WT Mice. (A) CCK-ir in HF of WT mice, shown at higher magnification in (E). In SNAP-25b deficient mice (B), CCK-ir disappears in PoDG and SLu, higher magnification in (F). (C) NPY-ir in WT HF, shown at higher magnification in (G). (D) Increased NPY-ir in PoDG, Mol, and SLu of SNAP-25b deficient (KO) mice, the latter at higher magnification in (H). Confocal micrographs of double-staining experiments taken from WT (I-K), and *neo*-excised SNAP-25b deficient (L) mice. CCK- (I) and SNAP-25-ir (J) in fibers/terminals of SLu in WT mice. Arrowheads in (J) point to strong SNAP-25^+^ fibers. (K) Double-staining of CCK- (green) and SNAP-25-ir (red) in WT SLu. (L) NPY-ir, lacking in fibers/terminals of WT SLu, is highly expressed in *neo*-excised SNAP-25b deficient mutants. In homozygous *neo*-excised SNAP-25b deficient mice, SNAP-25-ir fibers in SLu are often thicker (arrowheads in (L) compared to WT mice [(L) cf. (J,K)]. Insets in (K,L) show respective photomicrographs at higher magnification. (M) BDNF-ir in WT HF, the PoDG, and SLu shown at higher magnification in (O). (N) Increased BDNF-ir in PoDG and SLu of mutant mice, the DG shown at higher magnification in (P). (Q) Strong DCx^+^ cell bodies in SGZ of WT DG, shown at higher magnification in (S). (R) Increased number of cell bodies randomly distributed in SGZ and granular cell layer of *neo*-excised SNAP-25b deficient mutants, with higher levels of DCx^+^ fibers in Mol, shown at higher magnification in (T). Arrowheads in (T) point to DCx^+^ cell bodies in the granular cell layer. Cx, cortex; DG, dentate gyrus; LMol, lacunosum Mol; Mol, molecular layer of DG; Or, oriens layer of HF; PoDG, polymorph layer of DG; Py, pyramidal layer of HF; Rad, stratum radiatum of HF; SGrDG, supragranular layer of DG; SGZ, subgranular zone of DG; SLu, stratum lucidum of HF. Scale bars: (D = A–C = 500 µm), (H = E–G = 200 µm), (I = J–L = 50 µm), (K, inset = L inset = 10 µm), (M = N = 500 µm), (O = P = 200 µm), (Q = R = 100 µm), (S = T = 50 µm). WT (n = 2), SNAP-25b mutant mice (n = 2).

WT mice displayed a wide expression of CCK immunoreactivity (-ir) in the HF, being strongest in SLu and supragranular layer of the dentate gyrus (DG) (Figure 6A and 6E). Double-labeling experiments demonstrated that CCK^+^ terminals partially overlapped with SNAP-25-ir in SLu (Figure 6I–6K); however, SNAP-25-ir had a wider distribution in this layer. CCK-ir in SLu was virtually absent in homozygous *neo*-excised SNAP-25b deficient mutants (Figure 6B and 6F, c.f. Figure 6A and 6E), but was increased in cortex (Figure 6B). NPY^+^ fibers and interneurons were fairly evenly distributed in the WT mouse HF (Figure 6C), but NPY-ir was strongly increased in SLu and the polymorph layer of *neo*-excised SNAP-25b deficient mutants (Figure 6D and 6H, c.f. Figure 6C and 6G). NPY^+^ terminals/fibers in SLu of mutant mice overlapped with SNAP-25, although SNAP-25-ir had a more extensive distribution (Figure 6L). Diffuse NPY-ir was increased in the molecular layer of the DG, with lower levels in cortex of *neo*-excised SNAP-25b deficient mice (Figure 6C, c.f. Figure 6D). BDNF-ir was weak in SLu and polymorph layer of the WT (Figure 6M and 6O), but increased in mutant mice (Figure 6N and 6P). In WT mice, DCx^+^ cell bodies were distributed as a single layer in the DG subgranular zone (SGZ), while DCx^+^ processes had an even localization in the molecular and granular cell layers (Figure 6Q and 6S). The number of DCx^+^ cell bodies was increased by 140% in the SGZ of *neo*-excised SNAP-25b deficient mice, including cells migrating through the granular cell layer, with a large increase in DCx^+^ fibers in the molecular layer (Figure 6R and 6T, c.f. Figure 6Q and 6S and Text S1, Figure S3). No seizures were observed in juvenile *neo*-excised mutants prior to weaning, and in 3-week-old animals the expression of neuropeptides and BDNF did not differ compared to WT littermates (data not shown).

In conclusion, deletion of SNAP-25b results in dramatic alterations in neuropeptide levels in the HF, which together with increased levels of BDNF and DCx may provide an environment that promotes neuroprotection/neuroproliferation in response to disrupted synaptic transmission and seizure activity in *neo*-excised SNAP-25b deficient mutants.

## Discussion

The protein sequences of the two splice variants of SNAP-25, SNAP-25a and SNAP-25b, are remarkably well conserved in higher eukaryotes and demonstrate 100% amino acid identity in human, mouse and chicken [23],[26],[43]. Duplicated exon 5 sequences have also been described in bony fish [44] but not in cartilaginous fishes as the ray *Torpedo marmorata* or in the fruit fly *Drosophila melanogaster* [45]. The *Torpedo* SNAP-25 protein shares 81% and the *Drosophila* protein 61% amino acid identity with the mouse SNAP-25b protein, respectively [45]. Thus, the duplication of exon 5 in the *Snap25* gene must have occurred during early bony fish development more than 400 million years ago, when cartilaginous and bony fish divided and arose from the *Placoderms*, the first primitive jawed fishes [46]. Although bony fish appear to have rather immature brain structures, the sensory and motor coordinating functions are well developed. After the original duplication event, subsequent accumulation of mutations, presumably in both exon 5a and 5b, have resulted in the two similar but distinct SNAP-25 proteins present in all higher vertebrates.

We asked ourselves if the ancient duplication of exon 5, followed by mutually exclusive splicing, and resulting in increased protein diversity of the SNAP-25 protein was a prerequisite for the development of more intricate neuronal functions found in higher vertebrates. We now show that expression of SNAP-25b is important for early postnatal development and synaptic plasticity. Moreover, genetic elimination of SNAP-25b results in progressive morphological and neurochemical changes in CNS and impairment of cognitive function in adult animals. However, in brains of homozygous *neo*-containing SNAP-25b deficient mutants SNAP-25 protein levels were reduced by 20%, indicating that the *Tkneo* gene suppressed gene expression. Reduced SNAP-25 protein levels in absence of SNAP-25b are lethal, as demonstrated in the homozygous *neo*-containing SNAP-25b deficient mutants. The phenotype becomes evident already in the second PN week with characteristic tremors and increased activity, including hyperactive episodes and reduced growth; all signs of uncontrolled neurotransmitter and hormonal release. The *neo*-excised SNAP-25b deficient mutants, with restored levels of SNAP-25 expression, escaped the critical early PN period with minor defects. Thus, the increased vulnerability in *neo*-containing SNAP-25b deficient mutants is the result of both lack of the SNAP-25b isoform and reduced levels of protein, underlining the importance of a balanced expression of SNAP-25 isoforms.

### SNARE Complexes in Adult SNAP-25b Deficient Mouse Mutants Are Less Stable and Demonstrate a Different Cellular Localization Than Those in WT Mouse Brain

In the SNARE complex with syntaxin and VAMP, SNAP-25 contributes with two amphipathic α-helices to the four-helix-coiled-coiled SNARE structure [16]. The nine amino acids that distinguish SNAP-25b from SNAP-25a encompass a region in the SNAP-25 protein that spans the last part of the N-terminal SNARE motif and the first part of the linker that separate the N- and C-terminal α-helices. Substitutions of two non-conservative amino acid differences in the SNAP-25a protein to resemble the SNAP-25b sequence followed by transient overexpression in mouse chromaffin SNAP-25 deficient cells changed the secretion capability of SNAP-25a to become similar to that of SNAP-25b [47]. In addition, it has been established that SNAP-25b containing SNARE complexes are more stable than those containing SNAP-25a [16], and that SNAP-25b when overexpressed, is more efficient in driving fusion of catecholamine-containing vesicles from chromaffin cells [28]. We took advantage of the fact that homozygous *neo*-excised SNAP-25b deficient mutants only express SNAP-25a instead of predominantly SNAP-25b as in adult WT mouse brain. We have now been able to show for the first time that *ex vivo* SNARE complexes in brain homogenates from *neo*-excised SNAP-25b deficient mutants dissociated at lower temperatures than WT SNARE complexes (mostly holding SNAP-25b). In this respect our attention was drawn to a SNAP-25b mouse mutant named the “blind-drunk mouse” (Bdr) with retinal degeneration (caused by choice of the background strain) and a mild phenotype with ataxia and impaired sensorimotor gating due to a dominant mutation in SNAP-25b [48]. The Bdr mouse expresses a SNAP-25 protein with even higher affinity for syntaxin than WT SNAP-25b. The SNAP-25b(Bdr) SNARE complexes, apparently more stable than complexes with WT SNAP-25b, result in impairment of both spontaneous and evoked release from cortical neurons and lack of facilitation during trains of high-frequency stimulation [48]. The outcome from studies of SNAP-25b(Bdr), SNAP-25b and SNAP-25a containing complexes suggest that the difference in secretory phenotype might be dependent on the difference in complex-stability; and possibly on the interaction with accessory factors due to the presence of different amino acids exposed on the surface of the N-terminal amphipathic α-helix.

SNAP-25a and SNAP-25b also exhibit differences in the linker region between the N- and C-terminal SNARE motifs, where a quartet of cysteines, implicated in membrane anchoring of the protein, exists in two different contexts [23]-[25]. We hypothesized that a difference in plasma membrane association between SNAP-25a and SNAP-25b also could add to the phenotype observed in our mutants. Therefore we performed sucrose density gradient fractionation of brain homogenates from adult *neo*-excised SNAP-25b deficient mutants (only expressing SNAP-25a) and WT littermates (predominantly expressing SNAP-25b). In agreement with our assumption the mutants only had around 80% of the SNAP-25 protein levels found in WT plasma membrane fractions. Instead, in mutant brain more SNAP-25 was detected in low-density fractions, representing soluble protein and/or protein associated with small intracellular organelles. This difference in subcellular localization of the two SNAP-25 isoforms might contribute to the phenotype of the mutants. Whether that depends on the ability of the two isoforms to associate with membranes due to the altered organization of the cysteine residues that are substrates for palmitoylation, or, whether SNAP-25a and SNAP-25b reside in complex with additional secretory factors that guide the subcellular localizations needs to be further investigated.

### Short-Term Synaptic Plasticity Is Impaired in SNAP-25b Deficient Mice

To explore a possible effect on presynaptic mechanisms we studied PPF at Schaffer collateral-CA1 pyramidal synapses. We observed a reduction of PPF in young *neo*-containing and *neo*-excised SNAP-25b deficient mutants during low-frequency stimulation (0.2 Hz), suggesting a specific effect of SNAP-25b-deficiency. During higher frequency stimulation (0.5 Hz), when Ca^2+^ may be expected to be elevated during longer time periods in the stimulated presynaptic terminals, a reduction of the PPF-ratio was observed in *neo*-containing experiments while there was only a non-significant tendency to a reduction in *neo*-excised SNAP-25b deficient mice. Whether this is a result of the presumed higher average Ca^2+^-concentration or due to other factors will need to be investigated in future studies.

Normally, only few synaptic vesicles are “fusion-competent” and require no further modifications prior to exocytosis. Remaining vesicles close to the site of fusion need to be “primed”, that is undergoing ATP- and Ca^2+^-dependent maturation steps in order to be mobilized to the fusion-competent state [49]. It has been demonstrated that there is a 2–3 fold higher ability of SNAP-25b to keep vesicles in the primed state than SNAP-25a, a feature not due to facilitated priming but rather dependent on a lower de-priming rate [28],[50],[51]. Furthermore, SNAP-25b has been suggested to sustain the exocytotic bursts in a more efficient way than SNAP-25a in flash photolysis experiments with caged Ca^2+^ in chromaffin cells [28]. The results from the above mentioned investigations indicate that SNAP-25 is not only involved in the membrane fusion reaction but is also likely to play regulatory roles prior to exocytosis, such as in mobilization, docking and priming of vesicles [28],[38],[47]. Thus, the results from our PPF studies on SNAP-25b deficient mutants may represent an effect of reduced stability and availability of primed vesicles and are in line with the suggested weaker exocytotic bursts by synapses with SNAP-25a-containing SNARE complexes. Interestingly Bronk *et al*. [38] showed that there is no synaptic facilitation in the few cultured SNAP-25 KO hippocampal neurons that respond to extracellular stimulation. It may be that the two isoforms of SNAP-25 differ in their ability to sustain Ca^2+^-dependent types of facilitation including PPF. At physiological levels of SNAP-25 expression and elevated Ca^2+^-concentrations the replacement of SNAP-25b by SNAP-25a does not affect synaptic release enough to result in a statistically significant effect. However, there was a tendency to a reduction that needs to be investigated further.

### Elimination of SNAP-25b Expression Impairs Cognitive Function, Triggers Morphological Changes, and Induces Neuroprotective Mechanisms

SNAP-25b is normally highly expressed in brain areas involved in cognitive function, such as the HF. Therefore we examined mutants in behavioral tasks dependent on hippocampal mechanisms. *Neo*-excised SNAP-25b deficient mutants demonstrated a higher anxiety index compared to WT mice, however, without alterations in overall locomotor activity in the elevated plus maze. This finding is consistent with the profound thigmotaxis observed in the computerized measurements obtained in the water maze task. In the Morris water maze task, homozygous *neo*-excised SNAP-25b deficient mutants were severely impaired in their ability to acquire the spatial learning task. This impairment cannot simply be explained by visual or motor disturbances although there was a trend for female *neo*-excised SNAP-25b deficient mutants to perform less well in the visible platform test. The failure of the mutants to consistently improve their performance in the visible platform test is probably related to the same mechanisms as those underlying the deficiency in spatial learning. It is important to stress that the mutants did not display impaired swim performance since they did not differ from WT in their swim speed. However, unlike the WT mice, the mutants displayed a high level of thigmotaxis in the water maze task. This suggests that the deficiency in spatial learning in the mutants could partly be related to their anxious phenotype evidenced by their profound thigmotaxis and/or related to attention deficits observed in the visible platform test.

Both *neo*-containing and *neo*-excised SNAP-25b deficient mutants demonstrate spontaneously occurring convulsive seizures and freezing behavior. The *neo*-containing SNAP-25b mutants are most severely affected with frequent attacks debuting around PN12-13, whereas in *neo*-excised SNAP-25b deficient mutants seizure activity is rare prior to young adulthood. These behavioral impairments were paralleled by increasingly pronounced anatomical and immunohistochemical changes, which are only observed after the debut of seizure activity. In *neo*-excised SNAP-25b deficient mice, mossy fibers appear notably enlarged and swollen with certain areas almost devoid of synaptophysin immunoreactivity, suggesting a locally decreased density of functional nerve endings. Increased NPY expression in mossy fibers after seizures is believed to be neuroprotective by dampening excitatory activity, while BDNF can regulate NPY expression [52],[53]. Both NPY and BDNF have been suggested to promote dentate SGZ neurogenesis [54],[55], and their elevated expression may contribute to the increase in DCx^+^ migrating neuronal precursor cells observed in our mutants. It has been reported that cells generated in the adult dentate gyrus mature into functional neurons that integrate into hippocampal circuitry [55],[56], and it was recently shown that newly generated neurons after epilepsy exhibit dampening characteristics [57]. Taken together, it is conceivable that pathology develops in the SNAP-25-expressing mossy fiber area in response to aberrant presynaptic plasticity and synaptic contacts. However, a detailed ultrastructural study will be required to determine any alterations in synaptic morphology.

### Conclusions

We here demonstrate the physiological importance of the ancient exon 5 duplication in the *Snap25* gene. The functions of SNAP-25a and SNAP-25b appear to complement each other in tuning the presynaptic exocytotic machinery towards different modes of release. For complex neuronal circuitries this modulation of release is necessary, and is also instrumental in protecting highly plastic brain areas from accumulating degenerative morphological changes with age. The development of higher brain functions during evolution has been complemented with expanding number of gene products, sometimes by gene duplications but primarily by increasing protein diversity and complexity via alternative splicing. The importance of alternatively spliced isoforms from other genes has previously been extensively analyzed *in vivo*, by hindering expression of selected exons using gene targeted mouse models [58]. This is the first time an exon knock-out/knock-in of tandem duplicated exons has been performed. We specifically investigated the physiological importance of removing one isoform while preserving total expression levels of the protein by a simultaneous knock-in of an extra copy of the remaining exon variant.

Interestingly, a growing amount of reports are connecting SNAP-25 function with a wide variety of behavioral and neuropsychiatric disorders, as well as linking it with cognitive capability in humans [29]–[32]. Our present findings in the SNAP-25b deficient mouse mutants suggest that even small alterations in the strictly regulated temporal and anatomical expression of SNAP-25 isoforms could add to these variations.

## Methods

### Generation of SNAP-25b Mutant Knock-Out Mice

To generate SNAP-25b deficient mouse mutants a targeting vector was generated where the exon 5b, located downstream of exon 5a in the *Snap25* gene, was substituted with an additional exon 5a. A *Tkneo* gene surrounded by loxP repeats was introduced into the vector [34] (Figure 1A). Homologous DNA recombination in embryonic stem cells (ES cells) was performed using standard procedures [59]. Chimeras that demonstrated germline transmission were chosen for establishing SNAP-25b KO mouse lines (see Text S1). Three mouse lines, bred independently from each other for at least ten generations on C57BL/6NCrl (B6) mice, were termed “*neo-*containing” as the *Tkneo* gene inserted into the targeting vector was still present in the modified *Snap25* gene. Two of the *neo-*containing lines were crossed with the Protamine-Cre recombinase transgene Prm1Cre [34], resulting in *in vivo* excision of the *Tkneo* gene. The *neo-*excised lines were bred independently onto B6 background until congenic, and the PrmCre1 transgene crossed out. Genotyping was routinely performed by PCR and by Southern blotting when necessary. *In vivo* expression of the introduced chicken exon 5a was demonstrated by RT-PCR (Text S1). All animal breeding and studies were done in accordance with the guidelines from local ethical committees.

### RNA Analysis

Mouse brains, minus the cerebellum, were isolated after terminal CO_2_ anesthesia and frozen in liquid N_2_. Total RNA was isolated from brain (PN14-15) using the GenElute Mammalian Total RNA kit (Sigma). For quantification of total SNAP-25 mRNA; 1 µg RNA, 10 µµ of each primer and a trace of [α^32^P] dCTP (3000 µCi/mmol, PerkinElmer Life Sciences) were used in 25 µl reactions with the SuperScript III RT-PCR System (Invitrogen). Semi-quantitative RT-PCR was performed with 20 cycles of amplification and SNAP-25 and glyceraldehyde-3-phosphate dehydrogenase (GAPDH) primers in the same reaction (see Text S1 for primer sequences and PCR programs). The RT-PCR products were separated on an 8% polyacrylamide Tris-borate EDTA (TBE) gel that was dried, and detected using a phosphoimager (BAS-1500, Fujifilm). Signal intensities were quantified in Image Gauge V3.45 (Fujifilm). Determination of SNAP-25a/b mRNA ratio expression was essentially performed as described [26] (and Text S1). Statistical analyses were made using Wilcoxon's signed-rank test.

### Western Blotting

Mouse brains were homogenized in (in mM): 20 HEPES, 1 MgCl_2_, 250 D-sucrose, 2 EDTA and protease inhibitor cocktail (Roche Diagnostics GmbH), pH 7.4. For whole cell homogenates cells were lyzed with 1% NP-40 (Sigma) and 5 µg protein was run on 10% Tris-glycine/NU-PAGE gels (Novex, Invitrogen) followed by Western blotting. Primary antibodies used were a rabbit polyclonal antibody against SNAP-25 from Synaptic Systems (1∶20,000 dilution), a rabbit polyclonal anti-SNAP-23 (1∶1000, Synaptic Systems), mouse monoclonals anti-syntaxin 1, HPC-1 (1∶50,000 and 1∶100,000) and anti-α-tubulin, clone DM 1A (1∶45,000), both from Sigma. Secondary antibodies were horseradish peroxidase-conjugated anti-rabbit and anti-mouse immunoglobulins (IgGs) from Dako Corporation and Rockland. Statistical analyses were made using Wilcoxon's signed-rank test for paired data.

### Determination of Stability of SNAP-25a and SNAP-25b Containing SNARE Complexes

Pulverized adult WT and homozygous *neo*-excised SNAP-25 deficient (KO) mouse brain tissues were homogenized in buffer described for whole cell homogenates. SDS sample buffer [0.5M Tris-HCl (pH 6.8), 20% glycerol, 4% SDS, 10% 2-mercaptoethanol and 0.05% Bromophenol Blue] was added to equal amounts (20–40 µg) of WT and KO homogenate before heating treatment at 70, 75, 80, 85, 90 and 100°C (boiling), or kept at 4°C, for 20 min. Treated samples were immediately loaded and separated on 10% Bis-Tris NU-PAGE gels (Invitrogen). SMI81 antibody (1∶500,000) was used to detect immunoreactivity of bands migrating as ternary SNARE complex or solitaire SNAP-25 protein. To measure the grade of disassembly of heat-resistant SNARE complex in WT and SNAP-25b KO tissue, percentage monomeric SNAP-25 protein of total SNAP-25 in ternary complex at 4°C was calculated and compared with the value calculated at a certain temperature. All experiments were performed at least three times. Results were analyzed with unpaired Student's t-test.

### Histology and Immunohistochemistry

For histological analysis, mice were anesthetized with isoflurane and transcardially perfused with PBS followed by freshly prepared 4% paraformaldehyde in PBS. Organs were post-fixed overnight, dehydrated in graded ethanol and embedded in paraffin according to standard procedures. 4 µm sections were stained with hematoxylin-eosin or cresyl violet.

For immunohistochemistry of paraffin embedded tissue, sections were collected on Superfrost Plus slides, deparaffinized (xylene), dehydrated (ethanol) and boiled by microwaving for antigen unmasking (see also Text S1). Antibodies used were mouse anti-SNAP-25 (1∶750, SMI 81, Sternberger Monoclonals), rabbit anti-SNAP-25 (1∶100, Synaptic Systems), mouse anti-synaptophysin (1∶200, SVP38, Sigma), and anti-mouse IgG-Alexa 488 or anti rabbit IgG-Alexa 546 (1∶250, Molecular Probes).

Neuropeptide immunohistochemistry was performed as described [60] (see also Text S1). Antibodies used were mouse anti-SNAP-25 (1∶750, 1∶2,000 SMI 81, Sternberger Monoclonals), rabbit anti-SNAP-25 (1∶100, Synaptic Systems), mouse anti-synaptophysin (1∶200, SVP38, Sigma), chicken anti-BDNF (1∶200, Promega) and goat anti-DCx (1∶100, Santa Cruz Biotechnology). For tyramide signal amplification (TSA+, NEN Life Science Products) antibodies used were rabbit anti-CCK (1∶8,000), or rabbit anti-NPY (1∶3,000) (for details see Text S1). Corresponding secondary antibodies were HRP-swine anti-rabbit IgG (1∶200, Dako), FITC-donkey anti-chicken, Cy3-donkey anti-goat and Cy3-donkey anti-mouse (all 1∶100, and from Jackson ImmunoResearch Laboratories).

### Determination of Growth Plate Zones in Hind Limb

Hind limb specimens were fixed in 4% formaldehyde in phosphate buffer and embedded in paraffin. Sections, 4–5 µm thick, were stained with hematoxylin and eosin. Growth plates of tibia and femur were scanned using a Zeiss Axiovert 35M microscope fitted with a LSR Astro Cam type TE3/A/S digital camera. Images were analyzed using Concord software from Life Science Resources Ltd. The width of the different zones was determined at least in triplicate per section. Per sample, at least two cross sections were measured. Data was analyzed with unpaired Student́s t-test.

### Electrophysiology

PN12-16 mice were terminally anesthetized with 100% CO_2_ and sacrificed by decapitation. Brains were quickly removed and placed in ice-cold low Ca^2+^/ high Mg^2+^ artificial cerebrospinal fluid (aCSF) containing (in mM): 124 NaCl, 5 KCl, 1.24 NaH_2_PO_4_, 0.5 CaCl_2_, 10 MgSO_4_, 26 NaHCO_3_, and 10 glucose, and oxygenated with 95% O_2_/5% CO_2_ (pH 7.4). Coronal hippocampal slices (350 µm thick) were cut on a vibratome (Leica VT1000S, Leica Microsystems) and transferred to regular aCSF (same composition as above but with 2.4 mM CaCl_2_ and 1.3 mM MgSO_4_) at RT. Patch-clamp electrodes (6–10 MΩ) were filled with (in mM): 135 Cs-methane sulphonate, 10 HEPES, 1 EGTA, 4 Mg-ATP, 0.3 Na-GTP, 2–5 QX-314, and 8 NaCl (pH 7.25; osmolarity 270–280 mOsm). Whole-cell voltage clamp (−70 mV) recordings of evoked excitatory postsynaptic currents (EPSCs) were obtained at RT from CA1 pyramidal neurons of the hippocampus, visualized with differential interference microscopy (DIC) using an Olympus BX50WI microscope. 50 µM picrotoxin (Sigma-Aldrich) was added to the aCSF to block GABA_A_ receptor-mediated synaptic transmission. EPSCs were evoked by stimulation of Schaffer collaterals with fine concentric Pt/Ir bipolar stimulation electrodes (Fredrik Haer and Co.) placed in the stratum radiatum. Paired stimuli were delivered at IPIs from 40 to 300 ms at 0.2 and 0.5 Hz and data was collected after at least 5 minutes of baseline recording to make sure responses were stable. Statistical comparisons of PPF ratios between WT and SNAP-25b deficient mice were made with two-way repeated measurements analysis of variance (ANOVA). For additional information, see Text S1.

### Mouse Behavioral Analysis

The elevated plus maze analysis was performed as described previously [61]. For the water maze task, the animals were handled by the operator for a period of five days prior to the test and spatial learning and memory were examined as described before [62]. Locomotor activity was recorded by means of a multi-cage red- and infrared-sensitive motion detection system as described earlier for mice [61].

Data from the behavioral testing was analyzed by non-parametric statistics using Kruskal-Wallis ANOVA followed by Mann-Whitney U as the Post-hoc test.

## Supporting Information

Figure S1 Weight curves of *neo*-excised SNAP-25b deficient mouse mutants from PN5 to PN15. (A) Weight gain was not reduced for *neo*-excised male mutants compared to male WT littermates (n = 6 animals of each genotype). (B) *Neo*-excised female SNAP-25b mouse mutants at the second PN week weigh less than their WT littermates (**p<0.01, n = 6 females of each genotype). Data was analyzed with two-way repeated measures ANOVA.
(1.30 MB TIF)

Figure S2 Sucrose density gradient fractionation of brain homogenates from 2–5 months old *neo*-excised SNAP-25b deficient and WT mice for comparison of subcellular localization of SNAP-25a (KO) and SNAP-25b (WT) proteins. More SNAP-25a than SNAP-25b protein could be found in low-density fractions, as shown in the representative immunoblots. *Neo*-excised SNAP-25b deficient mice (KO) had 80.4±3.4% (*p = 0.05) SNAP-25 in plasma membrane (PM)-associated fractions using a polyclonal antibody, whereas monoclonal antibody resulted in 86.5±5.5% PM-associated protein (*p* = 0.1, n.s.). Mean±S.E.M., n = 3 for each genotype of animals, and each gradient was run in triplicate for each antibody, data was analyzed using Mann-Whitney U test.
(1.21 MB TIF)

Figure S3 Doublecortin-ir cells in the hippocampal dentate gyrus. Number of DCx+ migrating neuronal precursor cells was increased by 140% in the *neo*-excised SNAP-25b deficient (KO) mutants. Number of cells was 298±28 in the homozygous *neo*-excised SNAP-25b deficient KOs, compared to 125±20 in WT (two levels were counted from each animal and in both hemispheres, n = 2 animals of each genotype, Student́s t-test, mean±SD).
(1.39 MB TIF)

Text S1 Supplementary information.
(2.98 MB DOC)
