# Supplementary figures and images for: An Ancient Duplication of Exon 5 in the Snap25 Gene Is Required for Complex Neuronal Development/Function
Source: PLoS Genet. 2008 Nov 28;4(11):e1000278. doi: 10.1371/journal.pgen.1000278 (PMC2581893; doi:10.1371/journal.pgen.1000278)

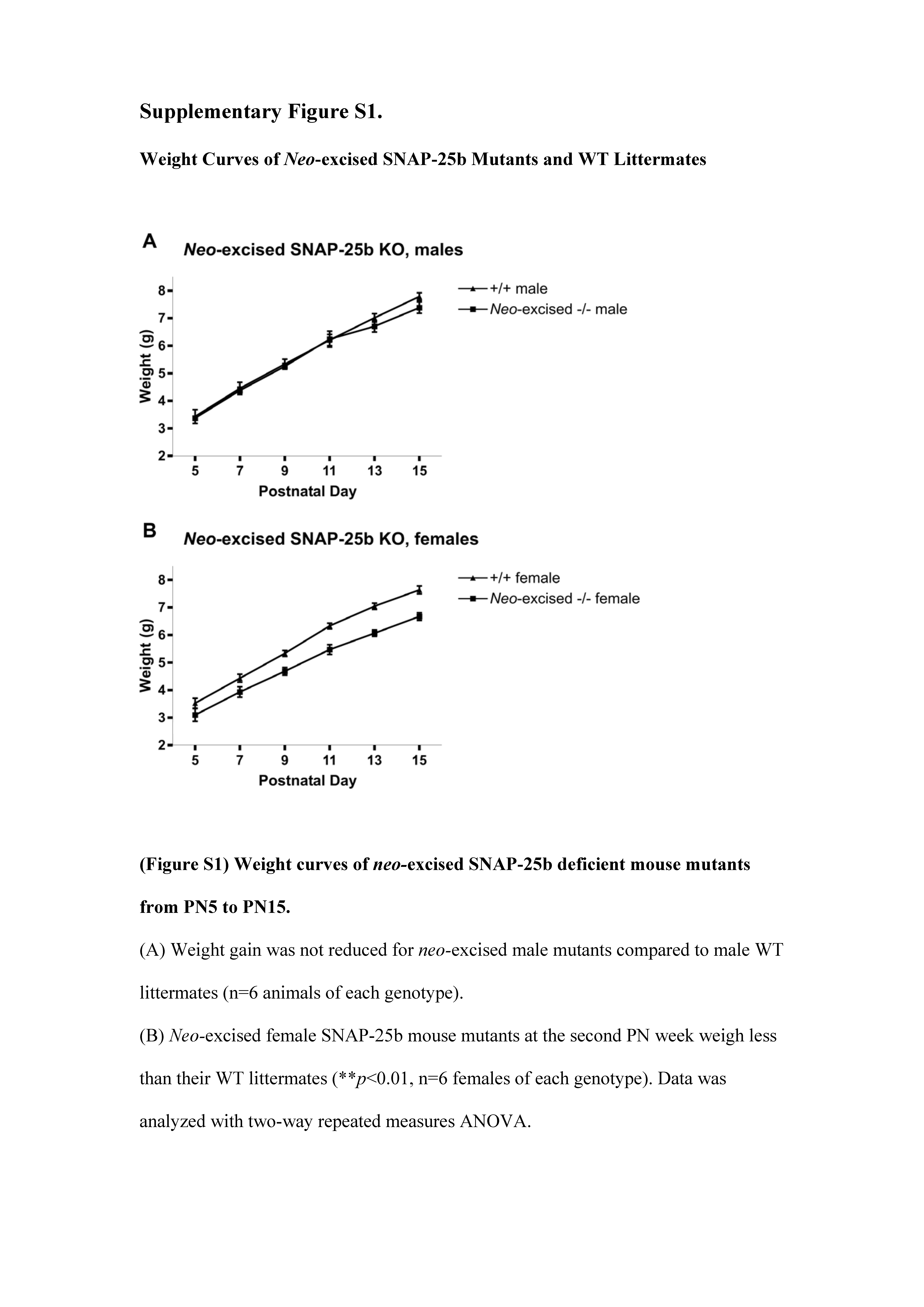

Supplement: Figure S1 — Weight curves of neo-excised SNAP-25b deficient mouse mutants from PN5 to PN15. (A) Weight gain was not reduced for neo-excised male mutants compared to male WT littermates (n = 6 animals of each genotype). (B) Neo-excised female SNAP-25b mouse mutants at the second PN week weigh less than their WT littermates (**p<0.01, n = 6 females of each genotype). Data was analyzed with two-way repeated measures ANOVA. (1.30 MB TIF) [file pgen.1000278.s001.tif]

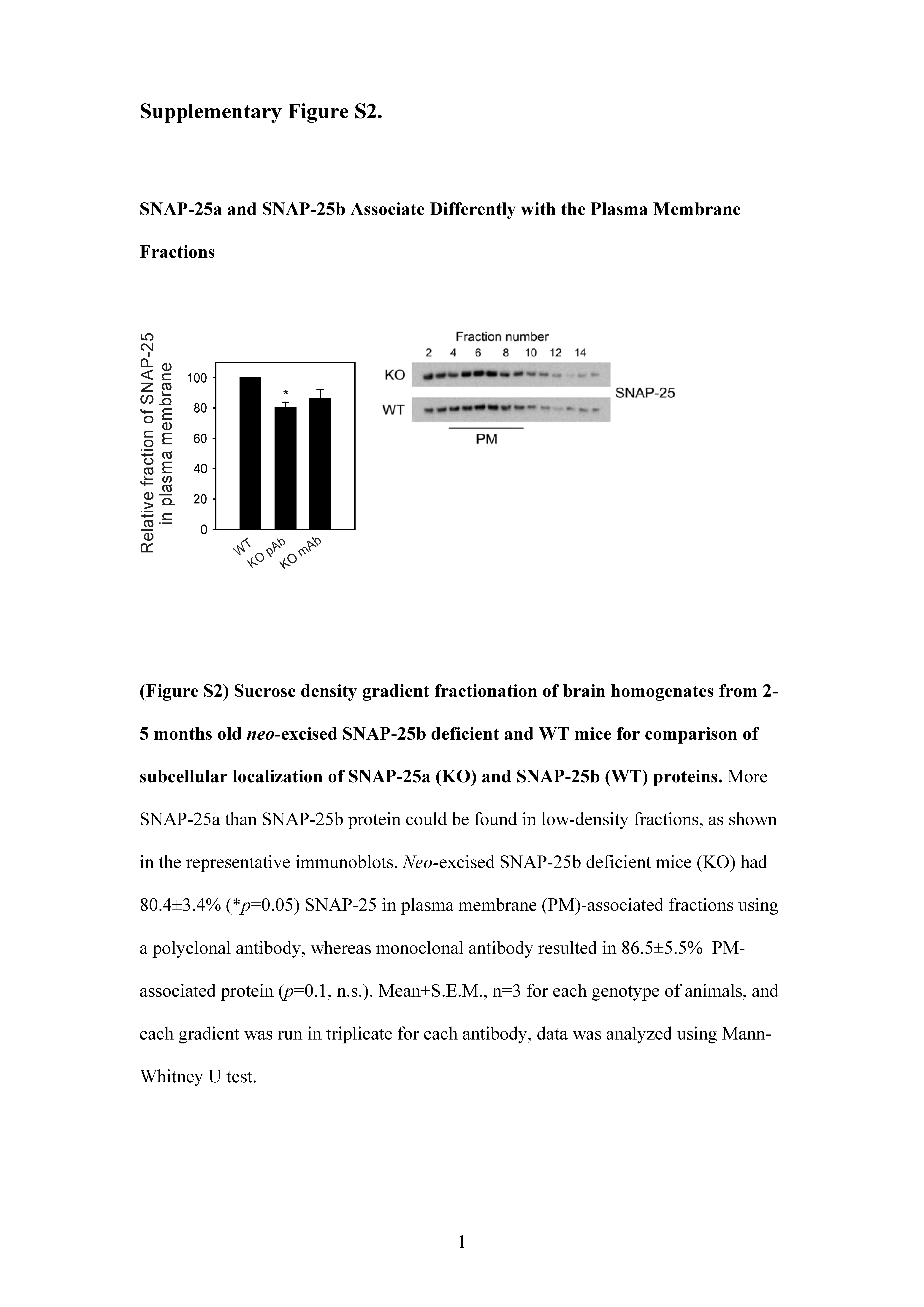

Supplement: Figure S2 — Sucrose density gradient fractionation of brain homogenates from 2–5 months old neo-excised SNAP-25b deficient and WT mice for comparison of subcellular localization of SNAP-25a (KO) and SNAP-25b (WT) proteins. More SNAP-25a than SNAP-25b protein could be found in low-density fractions, as shown in the representative immunoblots. Neo-excised SNAP-25b deficient mice (KO) had 80.4±3.4% (*p = 0.05) SNAP-25 in plasma membrane (PM)-associated fractions using a polyclonal antibody, whereas monoclonal antibody resulted in 86.5±5.5% PM-associated protein (p = 0.1, n.s.). Mean±S.E.M., n = 3 for each genotype of animals, and each gradient was run in triplicate for each antibody, data was analyzed using Mann-Whitney U test. (1.21 MB TIF) [file pgen.1000278.s002.tif]

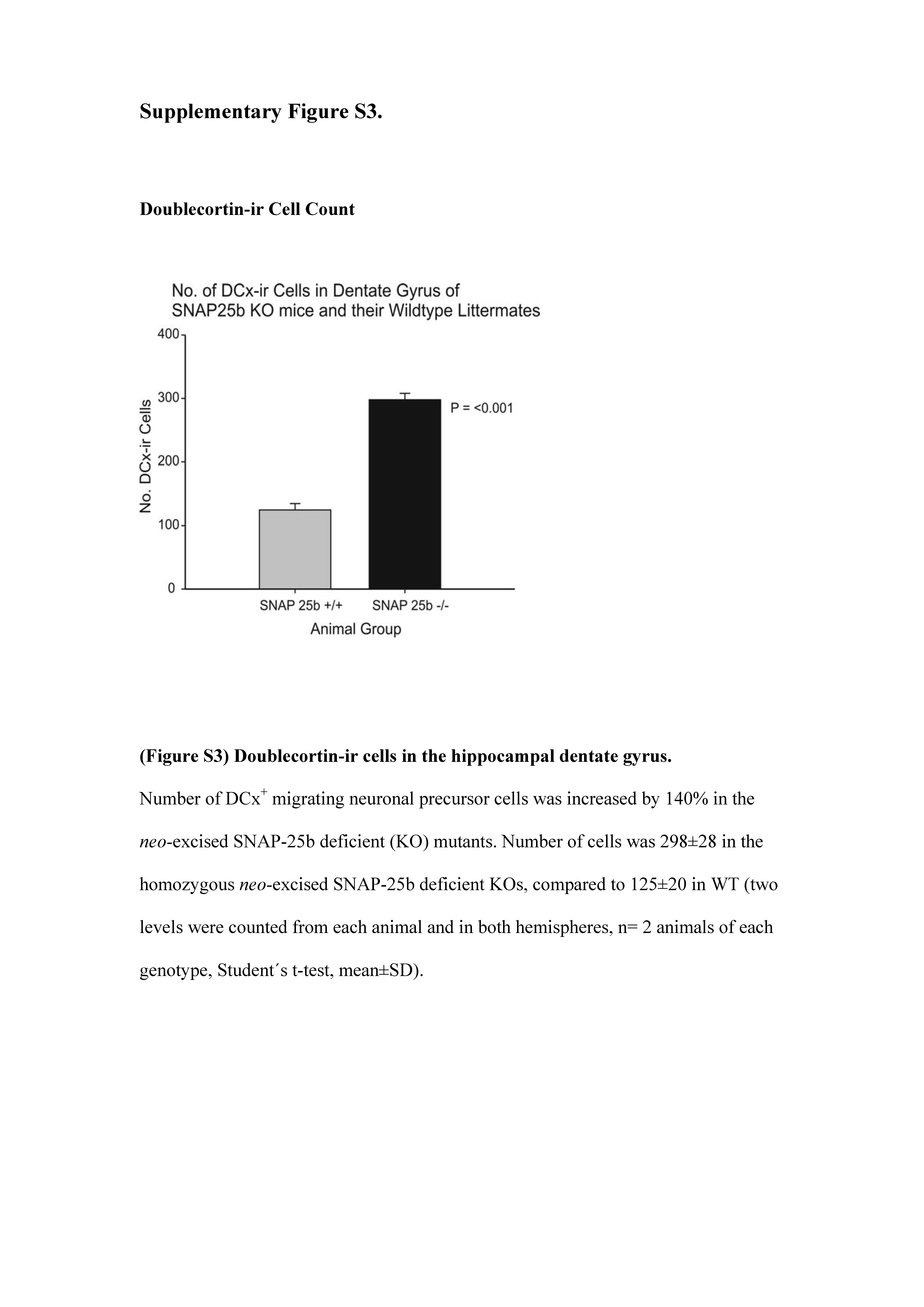

Supplement: Figure S3 — Doublecortin-ir cells in the hippocampal dentate gyrus. Number of DCx+ migrating neuronal precursor cells was increased by 140% in the neo-excised SNAP-25b deficient (KO) mutants. Number of cells was 298±28 in the homozygous neo-excised SNAP-25b deficient KOs, compared to 125±20 in WT (two levels were counted from each animal and in both hemispheres, n = 2 animals of each genotype, Student́s t-test, mean±SD). (1.39 MB TIF) [file pgen.1000278.s003.tif]
