# Supplementary material for: An Ancient Duplication of Exon 5 in the Snap25 Gene Is Required for Complex Neuronal Development/Function
Source: PLoS Genet. 2008 Nov 28;4(11):e1000278. doi: 10.1371/journal.pgen.1000278 (PMC2581893; doi:10.1371/journal.pgen.1000278)
Supplement: Text S1 — Supplementary information. (2.98 MB DOC) [file pgen.1000278.s004.doc]

**SUPPLEMENTARY INFORMATION**

**FIGURES**

**Supplementary Figure S1.**

**Weight Curves of *Neo*-excised SNAP-25b Mutants and WT Littermates**

**
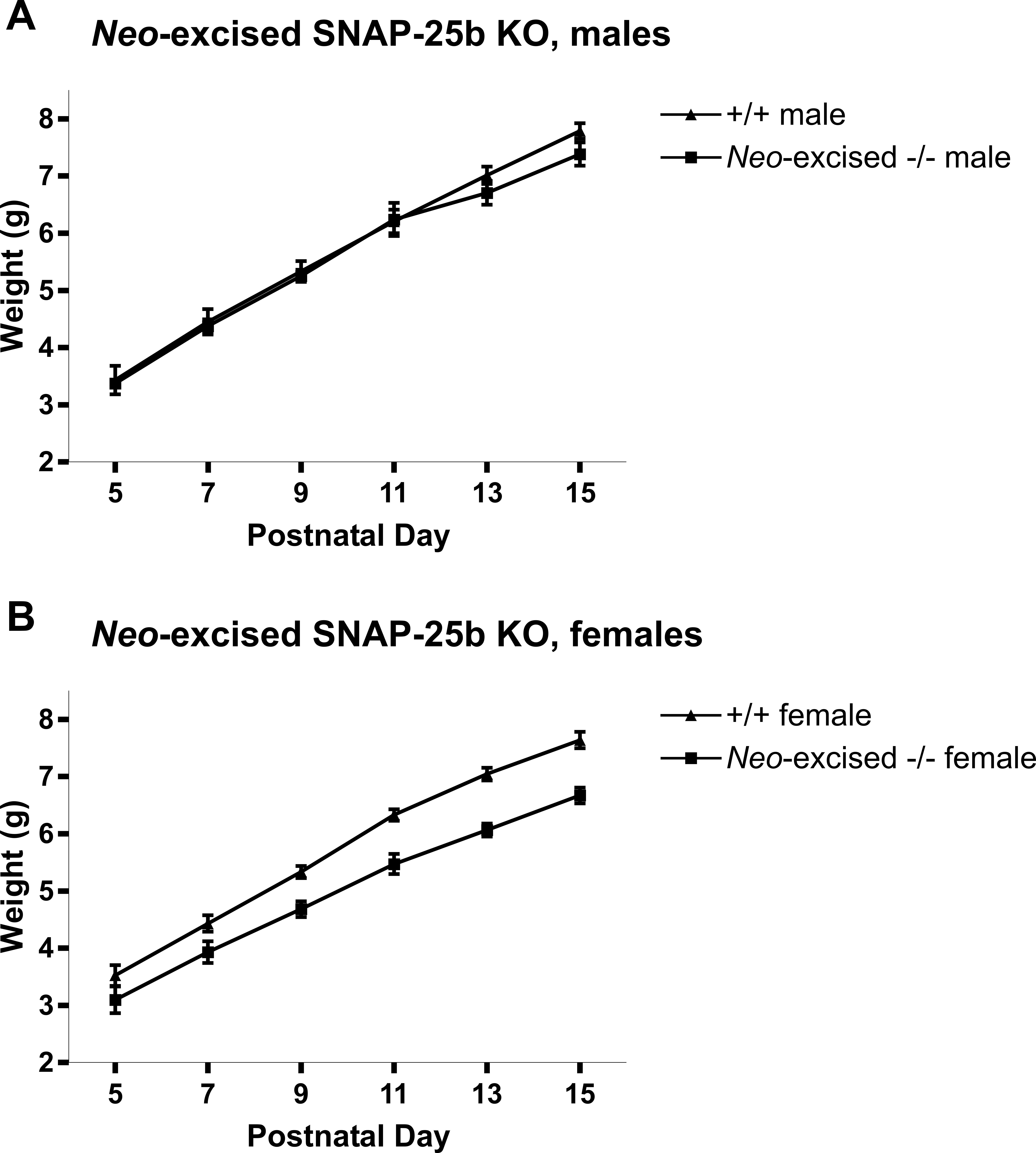
**

**(Figure S1) Weight curves of *neo-*excised SNAP-25b deficient mouse mutants from PN5 to PN15.**

(A) Weight gain was not reduced for *neo-*excised male mutants compared to male WT littermates (n=6 animals of each genotype).

(B) *Neo-*excised female SNAP-25b mouse mutants at the second PN week weigh less than their WT littermates (***p*<0.01, n=6 females of each genotype). Data was analyzed with two-way repeated measures ANOVA.

**Supplementary Figure S2.**

**SNAP-25a and SNAP-25b Associate Differently with the Plasma Membrane Fractions**

**(Figure S2) Sucrose density gradient fractionation of brain homogenates from 2-5 months old *neo-*excised SNAP-25b deficient and WT mice for comparison of subcellular localization of SNAP-25a (KO) and SNAP-25b (WT) proteins.** More SNAP-25a than SNAP-25b protein could be found in low-density fractions, as shown in the representative immunoblots. *Neo-*excised SNAP-25b deficient mice (KO) had 80.4±3.4% (**p*=0.05) SNAP-25 in plasma membrane (PM)-associated fractions using a polyclonal antibody, whereas monoclonal antibody resulted in 86.5±5.5% PM-associated protein (*p*=0.1, n.s.). Mean±S.E.M., n=3 for each genotype of animals, and each gradient was run in triplicate for each antibody, data was analyzed using Mann-Whitney U test.

**Supplementary Figure S3.**

**Doublecortin-ir Cell Count**


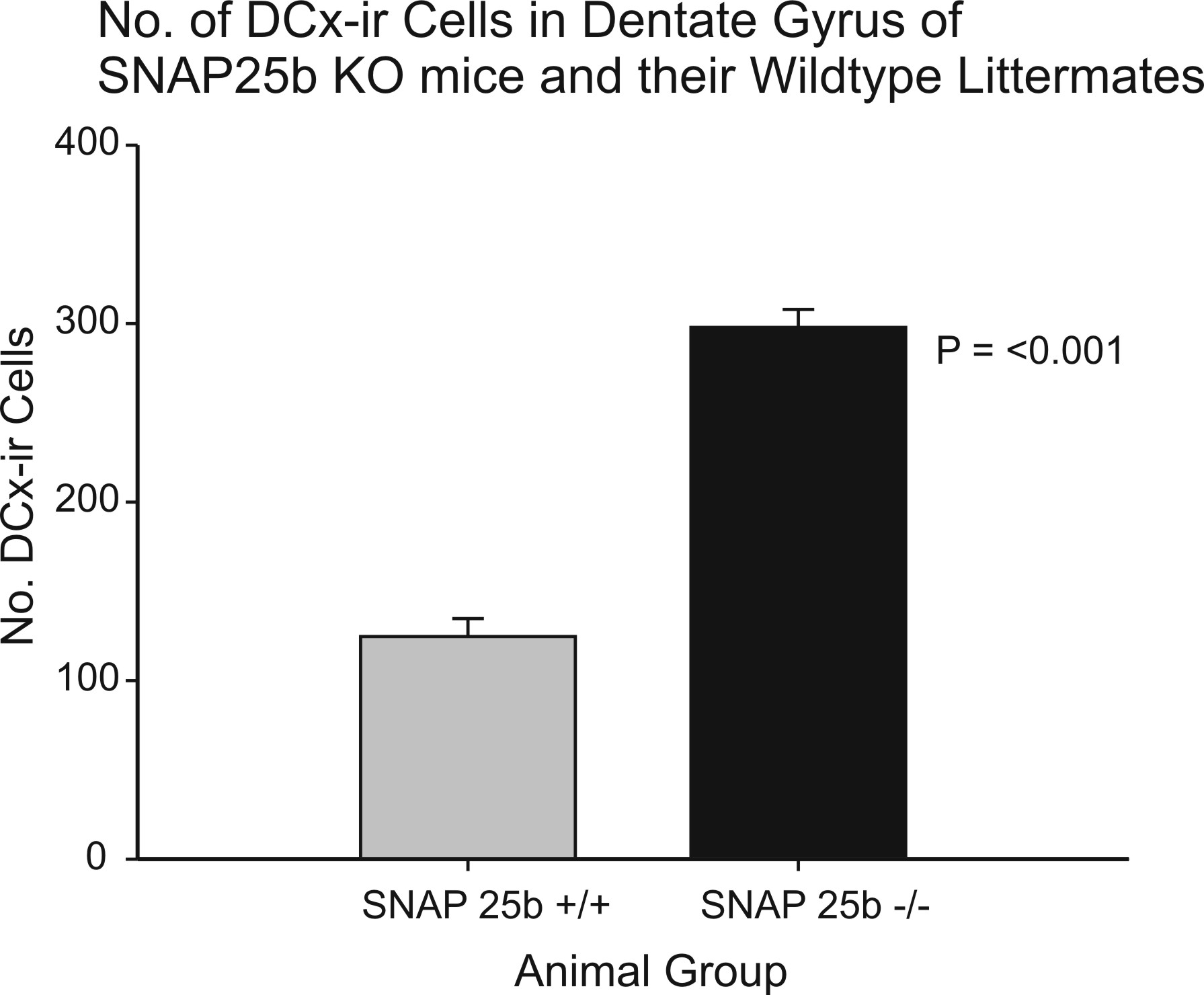


**(Figure S3) Doublecortin-ir cells in the hippocampal dentate gyrus.**

Number of DCx^+^ migrating neuronal precursor cells was increased by 140% in the *neo-*excised SNAP-25b deficient (KO) mutants. Number of cells was 298±28 in the homozygous *neo-*excised SNAP-25b deficient KOs, compared to 125±20 in WT (two levels were counted from each animal and in both hemispheres, n= 2 animals of each genotype, Student´s t-test, mean±SD).

**Supplementary Methods**

**Generation of SNAP-25b Mutant Knock-out Mice**

SNAP-25b knock-out mice were designed by generating a targeting vector where the exon 5b, located downstream of exon 5a in the *Snap*25 gene, was substituted with an additional 5a. Multiplex PCR was used to generate a recombinant fragment in which the mouse exon 5b was replaced with chicken exon 5a sequences using two genomic templates, a *PvuI*/*KpnI* fragment derived from the chicken gene encoding SNAP-25 that spans most of the 5a exon [1], and a mouse genomic *KpnI/StyI* DNA fragment containing most of the intervening sequence between exons 5a and 5b and the 5' part of the mouse exon 5b. The resulting PCR product was cloned as a *KpnI/HindIII* fragment into the vector pBluescript (Stratagene). DNA sequencing confirmed that this fragment contained the mouse intron between exon 5a and 5b downstream from the internal *KpnI* site, a chicken exon 5a replacing mouse exon 5b, and downstream sequences to the *HindIII* site located immediately 3' of the original exon 5b in the mouse gene. Thus, the only substitutions in the targeting construct were 31 of the 118 nucleotides in the mouse 5b exon coding sequence, resulting in an exon encoding the exon 5a protein domain. No nucleotides in surrounding non-coding sequences were changed, and the genetic modification introduced did presumably not cause any interference with the regulation of alternative splicing. Mouse genomic sequences used to construct series of intermediate subclones in the pBluescript KS II^+^ vector (Stratagene), and to build the final targeting vector were derived from a mouse *Snap25* genomic lambda phage clone, p4.2, as previously described [2]. The resulting construct, termed B52/loxP, spanned the mouse *Snap*25 gene sequences between an *XbaI* site located upstream of the original exon 5a and a *SacI* site located downstream of exon 6 in the mouse *Snap*25 gene. In the intron preceding exon 6, a *SalI/BglII* fragment spanning a *Tkneo* gene surrounded by loxP repeats was introduced [3]. The *Tkneo* gene was inserted in the same transcriptional orientation as the endogenous *Snap25* gene (see Figure 1A main text). Homologous DNA recombination was performed in totipotent embryonic stem cells (ES cells) using standard methods with positive selection with G418 for the presence of the *Tkneo* gene [4]. Two independent ES cell clones were identified that demonstrated homologous recombination and correct and expected replacement of the selected region of the *Snap*25 gene. These ES cell clones, #94 and #96, were injected into blastocysts and transferred into the uteri of pseudopregnant females at the **Karolinska Center for Transgene Technologies, Stockholm, Sweden. From 18 chimeras obtained, five were** tested for their ability to transmit the introduced mutation to the next generation by **mating with Charles River C57BL6/NCrl mice. Three males demonstrated germline transmission and became founders for three independent lines. Founder 1A and 3A were derived from ES-cell clone #96 and founder 2B from ES cell clone #94. These mouse lines are referred to as the** *neo-*containing **SNAP-25b mouse mutants as the selection cassette still was present in the modified *Snap25* gene.**

**Genotyping**

Genotyping was routinely performed by PCR analysis on DNA extracted from ear biopsies obtained when animals were ID-marked at weaning. DNA was extracted using the GenElute Mammalian Genomic DNA Miniprep Kit (Sigma-Aldrich) and approximately 50 ng of DNA was used for PCR assays. Genotyping of SNAP-25 animals was performed using a semi-quantitative PCR assay based on comparison of gene dosage ratio between the intact *Snap25* gene and the gene for *interleukin 1β* (*IL-1β* ) [5]. The primers recognizing SNAP-25 sequences were directed towards exon 5a and exon 5b and resulted in a 397 bp fragment and had the following sequences: forward 5a primer, 5’ – CGA AGA AGG CAT GAA CCA TAT CAA CC – 3’; reverse 5b primer, 5’ – TTG GTC CAT CCC TTC CTC AAT GCG T – 3’. The primers used for amplifying part of the *IL-1β* gene corresponded to sequences in exon 2 and exon 3, respectively. The primer sequences, amplifying a 602 bp fragment were: forward primer, 5’ – CCT GAA CTC AAC TGT GAA ATG CCA C – 3’; reverse primer, 5’ – GTC CGT CAA CTT CAA AGA ACA GGT C – 3’. PCR reactions (50 μl) contained: 0.5 mM NTPs, 0.25 units Taq Platinum (Invitrogen), PCR reaction buffer supplemented with 2.5 mM MgCl_2_ (Invitrogen) and 0.2 μM each of the primers. The PCR amplification was performed using the following program: 5 min at 94°C, followed by 7 cycles of denaturation at 94°C for 30 s, annealing at 50°C for 1 min and extension at 72°C for 1 min, followed by 25 cycles of denaturation at 94°C for 15 s, annealing at 50°C for 30 s and extension at 72°C for 30 s and ending with a final extension at 72°C for 7 min. The amplified PCR products, always run as duplicates, were separated by electrophoresis on a 1.0 % Tris-Acetate-EDTA (TAE) agarose gel and visualized by ethidium bromide staining. 1 Kb DNA ladder (Invitrogen) was used as size marker. When necessary, genotyping was also performed on tail DNA by Southern blotting using an *XhoI/PstI* 3’ flanking probe (see Figure 1A main manuscript). Genotyping of Prm1Cre mice using PCR was essentially performed as for the SNAP-25 animals except that 100-300 ng genomic DNA was used as template. The PCR program used was: 7 min at 94°C, followed by 35 cycles of denaturation at 94°C for 30 s, annealing at 53°C for 1 min and extension at 72°C for 1 min, followed by a final extension at 72°C for 7 min. The primer sequences were: forward, 5’ – CAC GAC CAA CTG ACA GCA AT – 3’; and reverse, 5’ – AGA GAC GGA AAT CCA TCG CT – 3’.

*Excision of the Tkneo gene.* Initially Southern blotting, as described above, was performed in order to establish that excision of the *Tkneo* gene was taking place when Cre-mice were mated with heterozygous SNAP-25b mutant animals. For further confirmation, a PCR genotyping protocol was used. Primer sequences and MgCl_2_ concentration were as suggested by the Jackson laboratory (0.75 μM and 2 mM, respectively). The primers for the *Tkneo* gene were: forward primer, 5’- CTT GGG TGG AGA GGC TAT TC – 3’, and reverse primer, 5’- AGG TGA GAT GAC AGG AGA TC – 3’. The PCR program was identical to that for the Cre primers, except for 55°C annealing temperature. All primers were purchased from Proligo, Sigma-Aldrich, and the PCR program was run on a GeneAmp PCR System 9700 from Applied Biosystems.

*The introduced chicken exon 5a is expressed in vivo in mutant mice.* In order to confirm that the introduced additional exon 5a, of chicken gene origin, was expressed in mutant mice, RT-PCR was performed on total RNA isolated from adult mutant mice brain and compared to WT littermates. Primers for detecting the two different SNAP-25a transcripts were: either, forward primer 5’ – ATG GCC GAA GAC GCA GAC ATG – 3’, reverse primer specific for mouse exon 5a, 5’ – TGA TAT GGT TCA TGC CTT CTT CG – 3’ and reverse primer specific for the genetically introduced chicken exon 5a, 5’ – TGA TAT GGT TCA TGC CTT CTT CA – 3’, or forward primer 5’ – ATG GCC GAA GAC GCA GAC ATG – 3’, reverse primer specific for mouse exon 5a, 5’ – TAT GAA AAG GCC ACA GCA TTT G – 3’ and reverse primer specific for the genetically introduced chicken exon 5a, 5’ – CAT ATG AAA AGG CCA CAG CAT TTT – 3’. The RT-PCR assays were based on the presence of two nucleotide differences between the mouse and chicken exon 5a sequences. The first set of primer combinations resulted in a PCR-fragment of 201 bp, and the second primer mix resulted in amplified products of 264 bp. The PCR fragments were analyzed on 2% agarose/TAE gels.

**RNA analysis primers and RT-PCR programs**

Primers for SNAP-25, spanning the whole SNAP-25 transcript, were: forward primer 5’ - CCC ACC ACT ACC ATG GCC GAG GAC – 3’, and reverse 5’ - CGG AAT TCT TAA CCA CTT CCC AGC ATC TT - 3´, giving a PCR product of 640 bp. For GAPDH the following primers were synthesized to yield a product of 208 bp: 5´ - GAC ATC AAG AAG GTG GTG AAG C – 3’ and 5’ - GTC CAC CAC CCT GTT GCT GTA – 3’. GAPDH primers were designed using cDNA sequences deposited in GenBank (accession numbers XM_147107 and X02231). The RT-PCR program used for total mRNA analysis was: 30 min at 55°C and 2 min at 94°C, followed by 20 cycles of denaturation at 94°C for 1 min, annealing at 55°C for 1 min and extension at 72°C for 1 min, and a final extension at 72°C for 7 min. For determination of SNAP-25a/b mRNA ratio the amplification steps in the RT-PCR were instead: 10 cycles of denaturation at 94°C for 30 s, annealing at 52°C for 30 s, and extension at 68°C for 1 min; then 24 cycles of amplification as before but the extension step was prolonged to 2 min.

**Sucrose Density Gradient Fractionation and Western blotting**

Mouse brains were homogenized in (in mM): 20 HEPES, 1 MgCl_2_, 250 D-sucrose, 2 EDTA and protease inhibitor cocktail (Roche Diagnostics GmbH), pH 7.4, followed by sucrose density gradient fractionation. 0.5 µg of sucrose gradient fractions was run on 10% Tris-glycine/NU-PAGE gels (Novex, Invitrogen) followed by Western blotting. Two primary antibodies against SNAP-25 were used, a mouse monoclonal, SMI81 from Sternberger Monoclonals (1:1,000 000) and a rabbit polyclonal from Synaptic Systems (1:25,000). Secondary antibodies were horseradish peroxidase-conjugated anti-rabbit and anti-mouse immunoglobulins (IgGs) from Dako Corporation and Rockland. Statistical analyses were made using Mann-Whitney U test.

**Immunohistochemistry**

For immunohistochemistry studies paraffin sections were collected on Superfrost Plus slides. The sections were deparaffinized using xylene, followed by ethanol. Sections were boiled by microwaving in 10 mM citrate buffer (Merck) pH 6.0 for 10 min for antigen unmasking, allowed to cool (1 h), and washed in Tris-buffered saline with Tween (TBS-T, pH 7.6) (used for all washing and incubation steps). Sections were blocked (5% goat serum), and incubated in mouse anti-SNAP-25 (1:750 SMI 81, Sternberger Monoclonals), rabbit anti-SNAP-25 (1:100, Synaptic Systems) or mouse anti-synaptophysin (1:200, SVP38, Sigma) for 16 h at 4°C, followed by anti-mouse IgG coupled to Alexa 488 or anti-rabbit IgG coupled to Alexa 546 (both 1:250, Molecular Probes) for 1 hr at room temperature (RT). Slides were mounted in Vectashield (Vector Laboratories). Specimens were observed using a Leica TCS-SP2-AOBS confocal laser-scanner equipped with Argon and HeNe lasers connected to a Leica DMLFSA microscope (Leica Microsystems Heidelberg GmbH), using a 488 and a 543 laser in sequential scanning mode.

For immunohistochemical analysis of expression of neuropeptides and some other markers, mice were anesthetized with isoflurane and perfused with Ca^2+^-free Tyrode’s buffer, followed by a mixture of 4% paraformaldehyde and 0.2 % picric acid in 0.16 M phosphate buffer [6,7]. The brains were dissected out, postfixed, frozen and sectioned (14 μm), following the same procedure as described previously [8]. The tyramide signal amplification (TSA+, NEN Life Science Products) immunohistochemical method [9] was used with rabbit polyclonal antisera raised against anti-CCK [10] (1:8,000), or rabbit anti-NPY (1:3,000; Theodorsson, unpublished). Incubation in primary antisera was followed by horseradish peroxidase-conjugated swine anti-rabbit IgG (1:200, Dako). Chicken antibody against anti-BDNF (1:200, Promega), goat anti-DCx (1:100, Santa Cruz Biotechnology), or mouse anti-SNAP-25 antisera (1:2,000, SMI 81, Sternberger Monoclonals) were also used, the latter for double-staining after TSA+ processing. Following incubations in BDNF, DCx or SNAP-25 primary antisera, sections were incubated at RT with FITC-conjugated donkey anti-chicken, Cy3-conjugated donkey anti-goat and Cy3-conjugated donkey anti-mouse, respectively (1:100, Jackson ImmunoResearch Laboratories), for 120 min at RT.

Sections were examined using a Nikon Eclipse E600 fluorescence microscope (Nikon), and photographs taken using a Hamamatsu ORCA-ER C4742-80 digital camera, using Hamamatsu photonics Wasabi 150 software. For confocal analysis, a Bio-Rad Radiance Plus confocal scanning microscope installed on a Nikon Eclipse E600 fluorescence microscope was used. FITC labeling was excited using a 488 nm argon laser and its signal detected using a HQ 530/60 (Bio-Rad) emission filter. For the detection of RRX and Cy3, the 543 nm HeNe laser in combination with HQ 590/70 (Bio-Rad) emission was used. Digital images were slightly modified to optimize for image resolution, brightness and contrast using Adobe Photoshop 7.0 software (Adobe Systems).

**Electrophysiology**

Brain slices were incubated for at least 1 h before being transferred to the recording chamber (volume = 2 ml) and perfused with regular aCSF (2-3 ml/min). Patch-clamp electrodes were prepared from borosilicate glass microcapillaries (Harvard Apparatus) using a two-stage puller (PP-830, Narishige). Patched cells were identified under visual guidance (DIC/infrared optics) and recorded using an Axopatch 200B patch clamp amplifier (Molecular Devices Corp.). Signals were low-pass filtered at 5 kHz and digitized at 20 kHz with a Digidata 1200 (Molecular Devices Corp.) using Clampex 7 software (Molecular Devices Corp.). DC square current pulses of 0.2 ms duration were generated by an isolated current source (SIU90, Cygnus Technology) and delivered through the stimulation electrode at low intensity, in order to activate as few presynaptic fibers as possible but yet achieve stable responses. Data analysis was performed with Clampfit software (Molecular Devices Corp.) and statistical analyses with GraphPad Prism 4 (GraphPad Software Inc.). PPF ratios were calculated by dividing the amplitude of the second EPSC by the amplitude of the first EPSC (PPF ratio=EPSC_2_/EPSC_1_) and are presented as means±S.E.M. The mean PPF ratios were determined from individual mean PPF ratios per cell and IPI, based on >15 individual paired EPSCs per IPI.

**References for the supplementary material**

1. Bark IC (1993) Structure of the chicken gene for SNAP-25 reveals duplicated exons encoding distinct isoforms of the protein. J Mol Biol 233: 67-76.

2. Washbourne P, Thompson PM, Carta M, Costa ET, Mathews JR, et al. (2002) Genetic ablation of the t-SNARE SNAP-25 distinguishes mechanisms of neuroexocytosis. Nat Neurosci 5: 19-26.

3. O’Gorman S, Dagenais NA, Qian M, Marchuk Y (1997) Protamine-Cre recombinase transgenes efficiently recombine target sequences in the male germ line of mice, but not in embryonic stem cells. Proc Natl Acad Sci USA 94: 14602-14607.

4. Mansour SL, Thomas KR, Capecchi MR (1988) Disruption of the protooncogene int-2 in mouse embryo-derived stem cells: a general strategy for targeting mutations to non-selectable genes. Nature 336: 348-352.

5. Telford JL, Macchia G, Massone A, Carinci V, Palla E, et al. (1986) The murine interleukin 1 beta gene: structure and evolution. Nucleic Acids Res 14: 9955-9963.

6. Pease PC (1962) Buffered formaldehyde as a killing agent and primary fixative for electron microscopy. Anat Rec 142: 342.

7. Zamboni I, De Martino C (1967) Buffered picric acid formaldehyde. A new rapid fixative for electron microscopy*.* J Cell Biol 35: 148A.

8. Stanić D, Brumovsky P, Fetissov S, Shuster S, Herzog H, et al. T (2006) Characterization of neuropeptide Y2 receptor protein expression in the mouse brain. I. Distribution in cell bodies and nerve terminals. J Comp Neurol 499: 357-90.

9. Adams JC (1992) Biotin amplification of biotin and horseradish peroxidase signals in histochemical stains. J Histochem Cytochem 40: 1457-1463.

10. Frey P (1983) Cholecystokinin octapeptide levels in rat brain are changed after subchronic neuroleptic treatment. Eur J [Pharmacol](javascript:AL_get(this, 'jour', 'Eur J Pharmacol.');) 95: 87-92.
